# A Dysprosium Complex with Two Quasi-Degenerate Easy Axes

**DOI:** 10.1021/acs.inorgchem.5c03279

**Published:** 2025-11-08

**Authors:** Carlo Andrea Mattei, Niki Mavragani, Alexandros A. Kitos, Matteo Briganti, Diogo A. Gálico, Muralee Murugesu, Mauro Perfetti

**Affiliations:** † Department of Chemistry Ugo Schiff, 9300 University of Florence, Via della Lastruccia 3, 50019 Sesto Fiorentino, Italy; ‡ Department of Chemistry and Biomolecular Sciences, 6363 University of Ottawa, Ottawa, Ontario K1N 6N5, Canada; # Department of Chemistry Ugo Schiff and INSTM Research Unit, 9300 University of Florence, Via della Lastruccia 3, 50019 Sesto Fiorentino, Italy

## Abstract

The synergistic use of synthesis, single crystal X-ray Diffraction, torque and SQUID magnetometry, luminescence, and *ab initio* calculations is presented here to unambiguously unfold the magnetic anisotropy of an apparently ordinary low-symmetry eight-coordinated Dy^III^ complex. Surprisingly, we found two low-lying states extremely close in energy, both displaying a strong easy axis anisotropy, tilted by ∼90°. Such a result has been rationalized, and its validity was extended upon targeted molecular modifications. These findings open new possibilities for the exploitation of such a kind of magnetic anisotropy in molecular materials.

## Graphical abstract

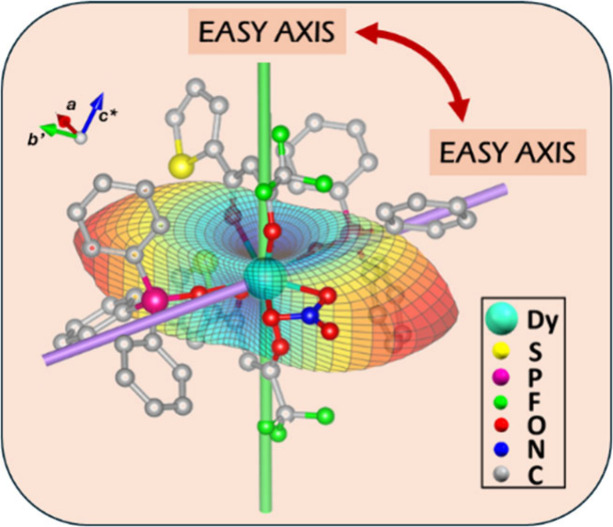

## Introduction

Magnetic anisotropy (MA) is the directional dependence of the magnetic properties of a material, and engineering its magnitude and shape is at the center of a vibrant research field.^1^ Arguably, the most desired MA is the easy axis type (*i.e.*, only one axis of the molecule can be easily magnetized) because it can be used to store information at the molecular level (single-molecule magnets), to shift the NMR signal and enhance structural resolution (pseudocontact shift agents), or even to cool a portion of space (rotating magnetocaloric effect).^2^ Since MA is primarily generated by strong spin–orbit coupling combined with a suitable crystal field (CF), lanthanide complexes have demonstrated unique potential as anisotropic building blocks.^3^ Among them, the Kramers’ ion Dy^III^ represents a convenient case for stabilizing large-*m*_*J*_ states, which favor an easy axis MA.^4^ This can be achieved in rather synthetically challenging low-coordinate complexes^5,6^ or by exploiting high-symmetry point groups.^7^ Conversely, high coordination numbers and low-symmetry environments generally induce a less predictable splitting accompanied by strong *m*_*J*_ mixing,^8^ hampering the possibility to control MA. However, this latter class of compounds usually benefits from relatively simple synthetic procedures and stability in air, desirable characteristics for the development of materials with practical applications. Therefore, we decided to explore the MA of air stable, easily synthesizable, and chemically tunable mononuclear lanthanide complexes. Specifically, we have focused on investigating β-diketonate ligands in combination with triphenylphosphine oxide and coordinating anions. In this paper, we report the synthesis the structural, and the magnetic investigation of the new complex [Dy­(TTA)_2_(NO_3_)­(TPPO)_2_] (**Dy**_**(TTA)**_; HTTA = 4,4,4-trifluoro-1-(2-thienyl)-1,3-butanedione, and TPPO = triphenylphosphine oxide (Figure 1)). Our study reveals that this molecule exhibits a very unusual MA: two orthogonal easy axes extremely close in energy. We suggest that the obtained MA could be used to achieve outstanding performance in the field of magnetic coolants.

**Figure 1:**
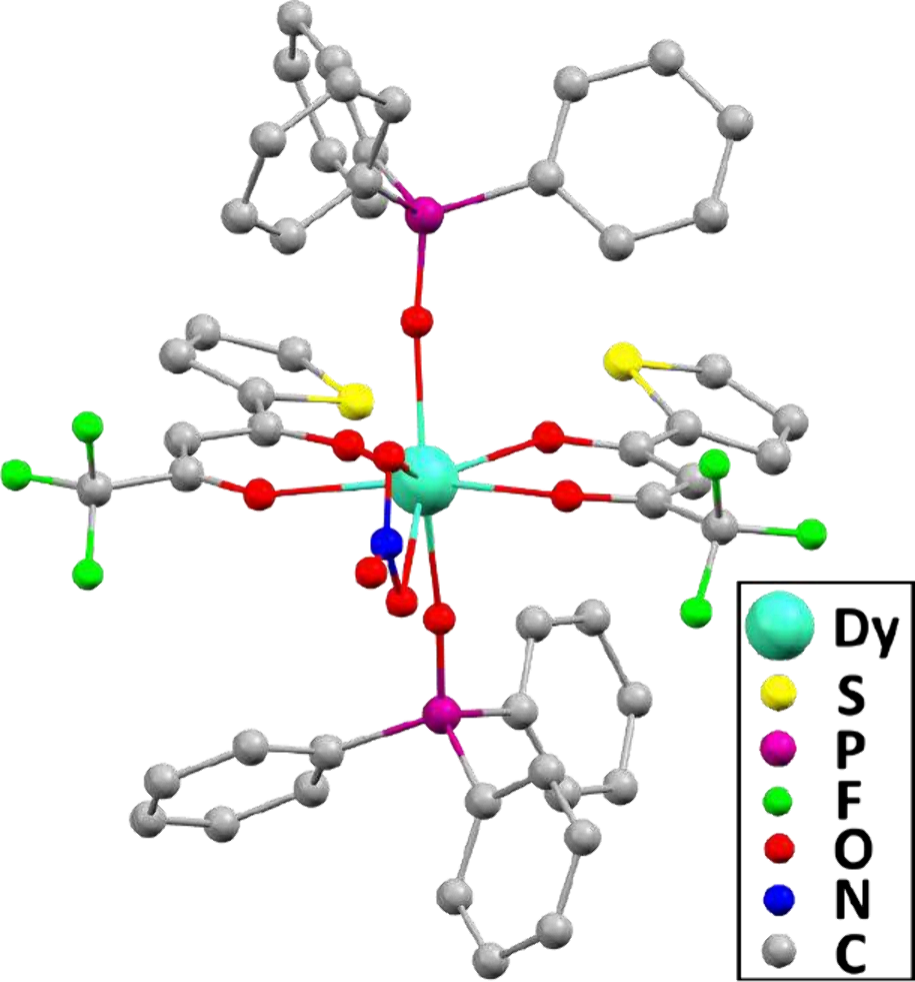
Representation of the SCXRD molecular structure of **Dy**_**(TTA)**_. Hydrogen atoms and disordered conformers have been omitted for the sake of clarity.

## Experimental Section

No uncommon hazards are noted. Detailed synthetic procedures are reported in the Supporting Information. All manipulations were performed under aerobic conditions.

Details regarding the experimental setup used for single crystal and powder X-ray diffraction, torque magnetometry, luminescence spectroscopy, thermogravimetric analysis, and dc/ac magnetometry measurements are reported in the Supporting Information, together with the procedure followed to perform the CASSCF calculations.

## Results and Discussion

Complex **Dy**_**(TTA)**_ crystallizes in triclinic *P*1 space group with one molecule in the unit cell, and its single-crystal X-ray diffraction (SCXRD) structure has been confirmed across a wide temperature range of 15–296 K. The Dy^III^ center is octacoordinated by four oxygen atoms from two TTA^–^ ligands, two oxygen atoms from two TPPO ligands, and two oxygen atoms from a nitrate anion. The thiophene rings of both TTA^–^ ligands were found to be disordered in 0.725(10):0.275(10) and 0.801(9):0.199(9) ratios by an approximate 180° rotation around the C–C bond linking the rings to the β-diketonate skeleton (Figure S2). Detailed structural analysis is provided in the Supporting Information.

To map its MA, we conducted a single-crystal torque magnetometry (TM) experiment by measuring the torque moment of a single crystal rotated in a static magnetic field (details in the Supporting Information). TM benefits from high sensitivity and the capability to disentangle noncollinear anisotropies.^9^ For collinear systems with *C*1 symmetry, like **Dy**_**(TTA)**_, the torque signal is usually simple to interpret. It vanishes every 90°, *i.e.*, when the projection of the easiest direction in the scanned plane is either parallel or perpendicular to the magnetic field.^10^ We performed three orthogonal rotations along the principal axes of the orthogonal reference frame ab′c* (Figure S8).

In two of these rotations (along *b*′ and along c*), the signal revealed the usual angular dependence (Figures S9 and S10), typical of several Dy^III^ complexes, suggesting an almost pure *m*_J_ = ±15/2 ground state with*g* ≈ (0 0 20). However, when the crystal was rotated along the *a* axis, the experimentally observed signal exhibited a more complicated shape, drastically changing upon variation of the temperature and magnetic field (Figure 2 and Figure S11). At a low temperature (2 K) and a high applied magnetic field (12 T), the measured torque exhibits four zero points (10°, 60°, 115°, and 150°). The shape of the signal resembles the one previously observed for an organometallic Er^III^ complex, where two noncollinear (orthogonal) molecules were present in the unit cell.^9^ Indeed, while a single *S* = 1/2 model cannot reproduce the data (Figure S12), a hypothetical highly axial (*g*_*z*_ = 20) two *S* = 1/2 spin system, where the two spins are tilted by approximately 70°, can simulate the experimental low-temperature torque curves (Figure S13). Symmetry-based considerations suggest that this scenario might be possible if the asymmetric unit cell contains two molecules linked by a *C*_2_ symmetry axis. To exclude the occurrence of a phase transition, the structure of the complex was acquired at 15 K and experimentally determined to still belong to triclinic *P*1 space group (Table S2). Moreover, the TM experiment was repeated several times by picking different indexed single crystals, confirming that the measured MA is intrinsic to the molecule.

**Figure 2:**
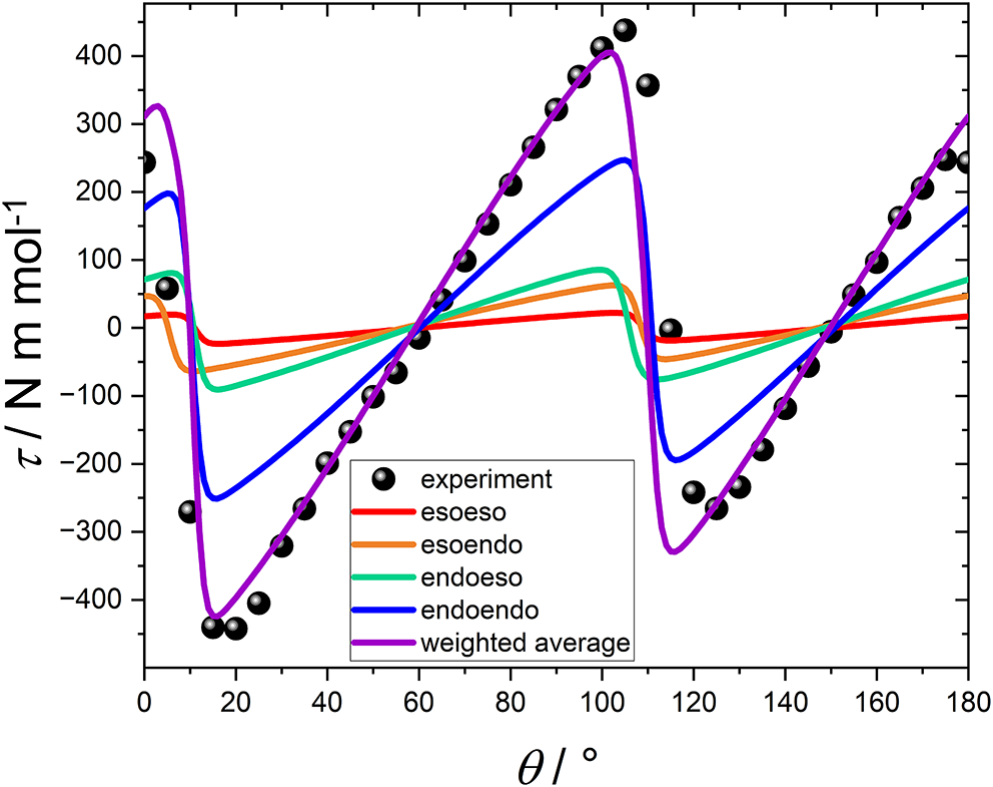
Angular dependence of the magnetic torque (τ) measured on a single crystal of **Dy**_**(TTA)**_ at 2 K and 12 T. The dots correspond to the experimental data. The lines correspond to the contribution from each isomer due to molecular disorder (red, orange, green, and blue) and the weighted averaged resultant (violet). See the Supporting Information for details.

The low symmetry of the complex makes the purely experimental determination of the CF parameters extremely hard.^11^ Therefore, we performed *ab initio* calculations at the Complete Active Space Self-Consistent Field (CASSCF) level of theory. The atomic coordinates were extracted from the SCXRD data structure collected at 100 K and considering the four isomers generated by the molecular disorder due to the thiophene rings on the β-diketonate. The energy ladder extracted from the calculations (Table S10) denotes that the two lowest doublets are separated by ∼5 cm^–1^, and both are significantly axial with *g*_*z*_ ≈ 19 (Table S11). Furthermore, the *z* axes of these two Kramers’ doublets are tilted by an angle of approximately 90°. The easy axis associated with the lowest doublet passes through the TPPO ligands, while the easy axis of the first excited doublet passes through the β-diketonate ligands.

To confirm the validity of the *ab initio* calculations, we probed the low-temperature luminescence of **Dy**_**(TTA)**_. Excitation of the sample at 365 nm and 1.8 K in the solid state promoted the efficient antenna transfer from the β-diketonate ligands to the Dy^III^ ion, allowing the observation of metal-centered luminescence emission (Figure S19). The spectrum, reported in Figure 3, shows more than the eight peaks expected for a single Dy center. This behavior, commonly observed in low-temperature lanthanide luminescence,^12–14^ is attributed to vibronic coupling. To confirm the nature of these additional features, we compared the ^4^F_9/2_ → ^6^H_15/2_ transition band with the Raman spectrum obtained at 293 K (Figure S20). It is possible to notice an excellent match between some of the Raman peaks and the additional features on the luminescence spectrum, confirming the vibronic nature of the additional peaks. It should be noted that some lines are slightly shifted, which is expected given the different temperatures (1.8 and 293 K) of each spectrum.

**Figure 3:**
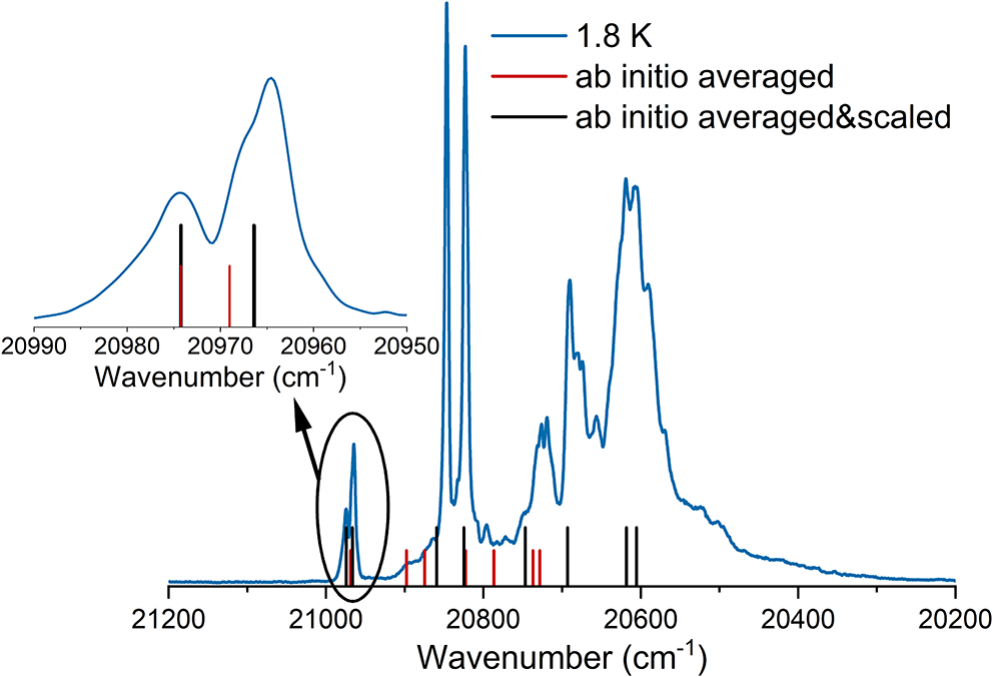
Emission spectrum at 1.8 K (blue) under 365 nm excitation of **Dy**_**(TTA)**_ in a solid state. The calculated (and weighted average for the molecular disorder) ^6^H_15/2_ energy splitting is depicted as red vertical sticks and as black vertical sticks when multiplied for a scale factor equal to 1.5, respectively (see the text).

The agreement between the luminescence data (^4^F_9/2_ → ^6^H_15/2_ transition) and the calculated ground level splitting is shown in Figure 3. It can be noticed that the *ab initio* calculations (red lines) considerably underestimates the experimentally determined magnitude of the CF splitting. Interestingly, if the calculated CF parameters are multiplied by a factor of 1.5 (Table S9), the agreement with the experimental data improves enormously, as previously observed.^15,16^ This semiempirical factor accounts for the lack of the ligand’s orbitals inside the active space, whose inclusion would be necessary to consider the partial covalent nature of the bonding between lanthanides and phosphine oxide ligands.^17^ Notably, the composition of the states does not vary significantly after the scaling. Therefore, we used this new set of parameters to fit and simulate the TM experimental data.

A weighted average of the torque produced by the four crystallographic isomers (based on the determined crystallographic ratios) is used to simulate the experimental torque. Notably, the thiophene ring rotation does not produce a significant change in the orientation of the easy axis (<5°, see Figure S14), making the inclusion of the four isomers correct, but not pivotal. Our simulation correctly reproduces the magnitude of the signal, but it is shifted by approximately 90° compared to the experimental data (Figure S14). This implies that the *ab initio*-calculated ground state is in fact the first excited state and *vice versa*; *i.e.*, the ground state easy axis lies along the β-diketonate ligands, as already observed for β-diketonate-based lanthanide complexes,^18–22^ and the first excited doublet passes through the TPPO ligands. This discrepancy is not surprising because correctly identifying the minima of the free energy of a system possessing two easy axes states so close in energy is extremely challenging, as already shown for the [DyDOTA­(H_2_O)]^−^ complex,^23^ where a subtle balance between Madelung potential and CF has to be taken into account. Indeed, a simple rotation of the reference frame correctly reproduces the torque curves at all fields and temperatures (Figure 2 and Figures S9–S11). Importantly, the same CF set is also used to satisfactorily simulate the field dependence of the magnetization and the temperature dependence of the magnetic susceptibility (Figures S21 and S22), further confirming the goodness of the model. No slow relaxation of the magnetization was detected for **Dy**_**(TTA)**_, suggesting the occurrence of rapid quantum tunnelling or relaxation via the first low-level excited state (Figure S23).

A visual representation of the anisotropic free energy of **Dy**_**(TTA)**_ at a low temperature and high fields superimposed with the molecular structure is reported in Figure 4. Details on how the plot was obtained can be found in the literature.^1,24^ Such a peculiar MA is generated because of the subtle balance between the CF imposed by the TTA^–^ and TPPO ligands. In a nutshell, the CF of our system is dominated by the four closest oxygen atoms (O(1), O(2), O(3) and O(5)), belonging to the TTA^–^ (O(3) and O(5)) and TPPO (O(1) and O(2)) ligands.

**Figure 4:**
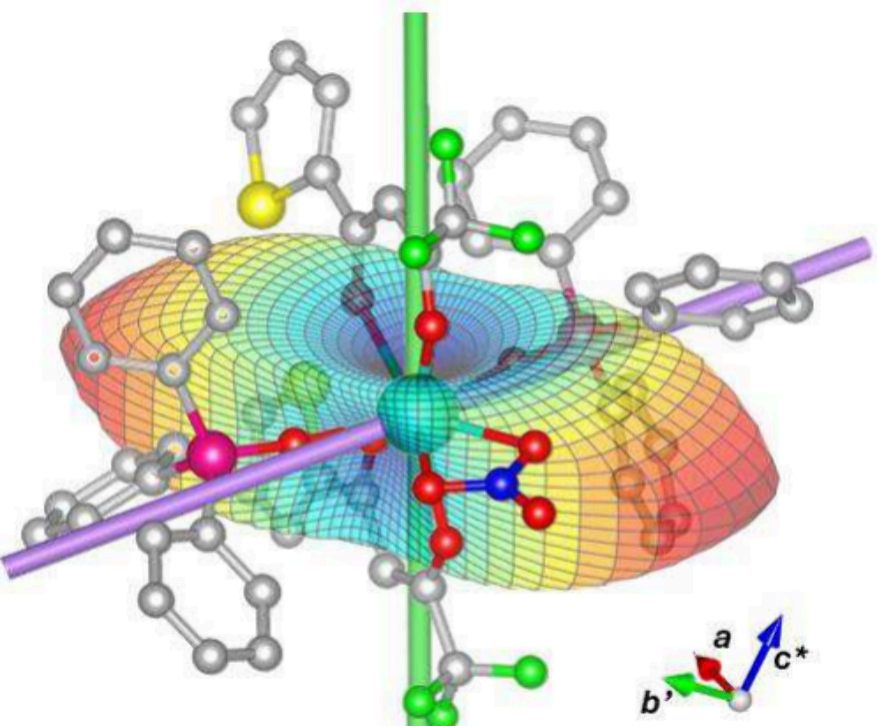
Superimposition of the **Dy**_**(TTA)**_ X-ray structure and its free energy simulated at 2 K and 12 T using the *ab initio*-determined CF parameters and the experimentally determined orientation. The easy axes of the lowest and first excited Kramers’ doublets are colored green and violet, respectively. The isotropic value of the free energy has been subtracted to emphasize the anisotropy of the free energy to which TM is sensitive.

Such a CF resembles other cases presented in the literature. If we consider the Dy–N direction as the unique (hard) axis of the structure, the CF can be seen as equatorial, which is quite similar to the case of the DOTA ligand.^25,26^ Another possible and convenient way to describe the CF of our system stems from recent literature on pentagonal bipyramidal Dy complexes.^27,28^ If the O(1)–Dy–O(2) axis is considered the unique axis and the nitrate ligand is considered monodentate, the structure can be seen as a distorted pentagonal bipyramid. In both cases, the main force determining the orientation of the ground state easy direction, and the gap between the ground and first excited states, is the competition between the TTA^–^ and TPPO ligands. The strongest interaction imparts the orientation of the easy axis, while the distortion generates a mixing of the states.^29–31^ In the complex discussed here, these interactions have comparable strength, generating two closely lying easy axes.

To further expand this concept, we exploited the amenability of both TTA^–^ and TPPO to easy chemical modification. To investigate the degree of tunability of such molecular architecture, we replaced the thiophene rings with phenyl rings using HBTA (4,4,4-trifluoro-1-phenylbutane-1,3-dione) instead of HTTA as the starting material. This procedure yielded theisomorphous complex [Dy­(BTA)_2_(NO_3_)­(TPPO)_2_] (**Dy**_**(BTA)**_), which also crystallized in triclinic *P*1 space group with one molecule in the unit cell (see the Supporting Information for experimental details, structural and thermogravimetric analyses). We focused on the crystal orientation, highlighting the presence of the two easy axes (b′c* plane scan). The experimentally observed zero torque points (Figure S16) suggest that the orientation of the ground state easy axis still lies along the β-diketonate ligands, as in **Dy**_**(TTA)**_. The shape of the signal (*i.e.*, zeroes every π/2) indicates that our chemical modification significantly increased the energy gap between the ground and first excited doublets. *Ab initio* calculations (Tables S14 and S15) performed on **Dy**_**(BTA)**_ correctly predict the orientation of the ground state easy axis (Figure S16). Again, we need to multiply the CF parameters by a factor of 2.2 to obtain an optimal agreement with the torque curves (Figure S16), suggesting that the *ab initio* method underestimates the gap between the ground and first excited states (18 and 39 cm^–1^ before and after scaling, respectively). In Figure S18, the free energy surfaces of **Dy**_**(TTA)**_ and **Dy**_**(BTA)**_ at a low temperature are compared, highlighting the presence of two minima (two closely lying easy axes) and one minimum (one easy axis), respectively.

The MA of **Dy**_**(TTA)**_ also offers exciting perspectives to engineer new materials, and we envisage its use in the field of cryogenics via rotating magnetocaloric effect experiments,^32^ where the easy axis nature of the ground state combined with a low-lying axial state could produce cooling powers far greater than those that can be obtained with conventional axial systems.^33^ Even though a real experiment is not possible due to the small crystal size, our model allows the simulation of the magnetic entropy change (−Δ*S*_R_)^2,34^ that a 90° rotation in the *z–x* plane (*i.e.*, easy axis to hard plane) of this complex could produce. Figure 5 shows the *–ΔS*_R_ values calculated for a highly axial Dy^III^ complex, *i.e.* *g* = (0 0 20) and a first excited state at a very high energy (black line), in comparison to the one expected for **Dy**_**(BTA)**_ (red solid line) and **Dy**_**(TTA)**_ (blue solid line) complexes. **Dy**_**(TTA)**_ displays the largest change in entropy with a maximum that is 1.6 times that of the hypothetical highly axial Dy^III^ complex. The rotating magnetocaloric performances of the **Dy**_**(TTA)**_complex make it competitive with other Dy-based molecular systems reported in the literature (Table 1).^33–36^

**Table 1:** Rotating Magnetocaloric Properties of Selected Molecules from the Literature

compound	max(Δ*S*_R_) (J kg^–1^ K^–1^)	applied field (T)	temperature (K)	experimental or calculated	ref	year
[Dy(TTA)_2_(NO_3_)(TPPO)_2_]	9.4	7	9.7	calculated	this work	2025
[Dy(BTA)_2_(NO_3_)(TPPO)_2_]	8.6	7	11.8	calculated		
*trans*-[CrF_2_(py)_4_][DyDOTA]	2.2	7	∼12.5	experimental	^29^	2024
	5		∼9	calculated		
[Dy(ZnL)_2_]CF_3_SO_3_	3.3	∼1	∼2	experimental	^30^	2022
	∼2.6	7	14			
[Dy(OSiMe_3_)_2_(4-Mepy)_5_(BPh_4_)]·0.5C_7_H_8_	3.05	5	19	experimental	^31^	2021
[{Dy(OAc)_3_(H_2_O)_2_}_2_]·4H_2_O	∼6	5	∼10	experimental	^28^	2016

**Figure 5:**
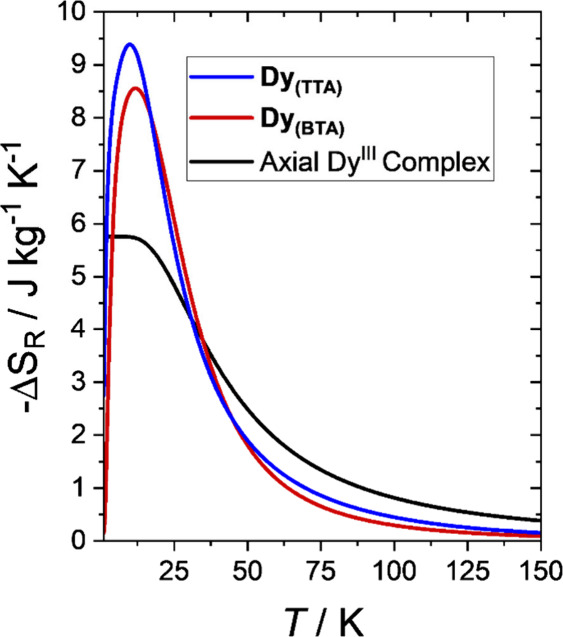
Magnetic entropy change calculated at *B* = 7 T for a highly axial Dy^III^ complex (black), **Dy**_**(BTA)**_ (red), and **Dy**_**(TTA)**_ (blue).

## Conclusions

In this work we reveal that the MA of an apparently ordinary dysprosium complex can be far more complicated and intriguing than expected. The presence of two orthogonal easy axes very close in energy was detected via torque magnetometry and further confirmed by luminescence and *ab initio* calculations. A simple ligand modification proved that the energy gap between the ground and first excited state can be chemically tuned. Finally, we show that, thanks to its unusual anisotropy, facile synthesis, and remarkable air stability, this molecular architecture holds great promise as an inexpensive, easy-to-make, and highly-performant magnetic refrigerant.

## Supplementary Material



## Notes

Data sets supporting this work (PDF)
