# Supplementary material for: A Dysprosium Complex with Two Quasi-Degenerate Easy Axes
Source: Inorg Chem. 2025 Nov 8;64(46):22648–53. doi: 10.1021/acs.inorgchem.5c03279 (PMC12648645; doi:10.1021/acs.inorgchem.5c03279)
Supplement: Supplementary file 1 [file ic5c03279_si_001.pdf]

Supporting information

A Dysprosium complex with two quasi-degenerate easy axes

Carlo Andrea Mattei,[a] Niki Mavragani,[b] Alexandros A. Kitos,[b] Matteo Briganti,[c] Diogo A. Gálico,[b] Muralee Murugesu [b]* and Mauro Perfetti [c]*

*m.murugesu@uottawa.ca *mauro.perfetti@unifi.it

[a] *Department of Chemistry Ugo Schiff, University of Florence, Via della Lastruccia 3, 50019, Sesto Fiorentino, Italy*

[b] *Department of Chemistry and Biomolecular Sciences, University of Ottawa, Ottawa, Ontario, K1N 6N5, Canada*

[c] *Department of Chemistry Ugo Schiff and INSTM Research Unit, University of Florence, Via della Lastruccia 3, 50019, Sesto Fiorentino, Italy*

Contents

1. General Methods and Materials……………………………………………………………………S2

2. Synthesis………………………………………………………………………………………………S2

3. Single-Crystal X-ray Diffraction……………………………………………………………………S3

4. Powder X-ray Diffraction…………………………………………………………………………S10

5. Thermogravimetric analysis………………………………………………………………………S10

6. Torque Magnetism…………………………………………………………………………………S11

7. Luminescence Spectroscopy………………………………………………………………………S21

8. Dc and ac Magnetic Studies………………………………………………………………………S24

9. Complete Active Space Self-Consistent Field (CASSCF) Ab Initio Calculations…………S26

10. References…………………………………………………………………………………………S31

1. General Methods and Materials

All manipulations were performed under aerobic conditions and all solvents and chemicals triphenylphosphine oxide (TPPO), 4,4,4-trifluoro-1-(2-thienyl)-1,3-butanedione (HTTA), 4,4,4-trifluoro-1-phenylbutane-1,3-dione (HBTA), and $Dy(NO_3)_3{\cdot}6H_2O$ were reagent grade and used without further purification. FT-IR spectra were recorded on a Nicolet Nexus 550 FT-IR spectrometer in the transmission window of 500 – 4000 cm^{-1}. Elemental Analysis was performed by Midwest Microlab.

2. Synthesis

Following a similar synthetic procedure to the previously published Eu and Tb analogues,[1–4] reaction of $Dy(NO_3)_3{\cdot}6H_2O$, TPPO, and HTTA or HBTA, in a 1:2:2 molar ratio in methanol (MeOH) afforded pale yellow solutions, that upon evaporation at room temperature, resulted in the formation of pale yellow prismatic crystals of the products.

$[Dy(TTA)_2(NO_3)(TPPO)_2]$ ($Dy_{(TTA)}$). In a 50 mL beaker, one equivalent of $Dy(NO_3)_3{\cdot}6H_2O$ (0.776 g, 1.7 mmol) was dissolved in MeOH (10 mL). A second 100 mL beaker was charged with two equivalents of TPPO (0.946 g, 3.4 mmol) and two equivalents of HTTA (0.755 g, 3.4 mmol), which were dissolved in MeOH (40 mL). The two solutions were slowly mixed, stirred for 10 minutes and filtered. After four days, pale yellow prismatic crystals were formed via slow evaporation of the mother liquor, collected by filtration, washed with cold MeOH and dried in air. Yield = 81%. FT-IR: 1622 ($\nu(C{=}O)_{TTA}$), 1535 ($\nu(C{=}C)_{TTA}$), 1460 ($\nu(P\text{-}Ph)_{TPPO}$), 1437 ($\nu(P\text{-}Ph)_{TPPO}$), 1410 ($\nu(C\text{-}S)_{TTA}$), 1161 ($\nu(C\text{-}F)_{TTA}$), 1136 ($\nu(P{=}O)_{TPPO}$), 1122 ($\nu(P{=}O)_{TPPO}$), 783 ($\nu(C\text{-}S)_{TTA}$), 744 ($\nu(Ph)_{TPPO}$), 721 ($\nu(P{=}O)_{TPPO}$), and 688 ($\nu(Ph)_{TPPO}$) cm^{-1}. Elemental Analysis: Calcd: C, 51.05 %; H, 3.13 %; N, 1.14 %, Found: C, 50.85 %; H, 3.23 %; N, 1.06 %.

$[Dy(BTA)_2(NO_3)(TPPO)_2]$ ($Dy_{(BTA)}$). In a 50 mL beaker, one equivalent of $Dy(NO_3)_3{\cdot}6H_2O$ (0.776 g, 1.7 mmol) was dissolved in MeOH (10 mL). A second 100 mL beaker was charged with two equivalents of TPPO (0.946 g, 3.4 mmol) and two equivalents of HBTA (0.735 g, 3.4 mmol), which were dissolved in MeOH (40 mL). The two solutions were slowly mixed, stirred for 10 minutes and filtered. After two weeks, pale yellow prismatic crystals were formed via slow evaporation of a MeOH/H_2O mixture, collected by filtration, washed with cold MeOH and dried in air. Yield = 79%. FT-IR: 1626 ($\nu(C{=}O)_{BTA}$), 1537 ($\nu(C{=}C)_{BTA}$), 1468 ($\nu(P\text{-}Ph)_{TPPO}$), 1437 ($\nu(P\text{-}Ph)_{TPPO}$), 1159 ($\nu(C\text{-}F)_{BTA}$), 1134 ($\nu(P{=}O)_{TPPO}$), 1121 ($\nu(P{=}O)_{TPPO}$), 762 (δ, C–H), 745

(ν(Ph)$_{TPPO}$), 721 (ν(P=O)$_{TPPO}$), and 689 (ν(Ph)$_{TPPO}$) cm^{-1}. Elemental Analysis: Calcd: C, 55.52 %; H, 3.49 %; N, 1.16 %, Found: C, 55.31 %; H, 3.50 %; N, 1.16 %.

3. Single-Crystal X-ray Diffraction

Crystals of **Dy$_{(TTA)}$** and **Dy$_{(BTA)}$** suitable for single-crystal X-ray diffraction (SCXRD) analysis were covered in parabar oil and mounted on MiTeGen cryoloop. Diffraction data (*ω*- and *φ*-scans) were collected on a Bruker AXS SMART (for **Dy$_{(TTA)}$**) or KAPPA (for **Dy$_{(BTA)}$**) APEX-II CCD single-crystal diffractometer (graphite monochromated Mo-Kα radiation, λ = 0.71073 Å), at room temperature and 100 K. For the single-crystal X-ray diffraction measurement at 15 K, an Oxford Diffraction Xcalibur3 CCD four-circle diffractometer with a graphite monochromator and Mo-Ka radiation, equipped with an Helijet cryostat, was used. The reflection intensities were corrected for absorption by using multi-scan of the SADABS[5] program. The structures were solved using direct methods with SHELXT[6] and refined by the full-matrix least-squares methods on F^2 with SHELXL-2018/3.[7] All H atoms were generated geometrically and were included in the refinement in the riding model approximation while their temperature factors were set to a multiple of the equivalent isotropic temperature factors of the parent site (see details in the CIF files). In the crystal structure of **Dy$_{(TTA)}$**, the thiophene rings of both TTA^{-1} ligands were found to be disordered in a 0.725(10) : 0.275(10) and 0.801(9) : 0.199(9) ratios (100 K) by an approximate 180° rotation of the rings around the C-C bond linking the rings to the β-diketonate units. A split site model was used along with soft restraints to bond lengths and displacement ellipsoids to model the disordered moieties (see details in the CIF file). The crystal of **Dy$_{(TTA)}$** was a non-merohedral twin and its structure was refined in two crystal domains (0.815/0.185) with the HKLF5 instruction. The crystal of **Dy$_{(BTA)}$** was a merohedral twin and its structure was refined as a 2-component inversion twin with a batch scale factor of 0.075. All geometric/crystallographic calculations were carried out using SHAPE,[8] PLATON,[9] and WINGX[10] programs while graphics were prepared with DIAMOND[11] and MERCURY.[12] The crystallographic data and structure refinement summary for **Dy$_{(TTA)}$** and **Dy$_{(BTA)}$** complexes are listed in **Table S1**.

Molecular and supramolecular features of Dy$_{(TTA)}$ and Dy$_{(BTA)}$ complexes

Both **Dy$_{(TTA)}$** and **Dy$_{(BTA)}$** crystallize in the triclinic *P*1 space group with one molecule in the unit cell (*z* = 1) (**Figure S1**). The single-crystal XRD structure of **Dy$_{(TTA)}$** was further confirmed in the temperature range 15-296 K (**Table S2**). In the crystal structure of **Dy$_{(TTA)}$**, the thiophene rings of both TTA^{-1} ligands were found to be disordered in a 0.725(10) : 0.275(10) and 0.801(9) : 0.199(9) ratio (100 K) by an approximate 180°

rotation of the rings around the C-C bond linking the rings to the β-diketonate units (**Figure S2**). The disorder slightly varies as a result of the experimental temperature or different crystals used for data collection. In $\mathbf{Dy_{(TTA)}}$, the Dy^{III} metal ion is coordinated to two chelating TTA^{-1} monoanionic ligands and two monodentate TPPO neutral ligands. The coordination sphere of the Dy^{III} ion is completed by one bidentate chelating NO_3^- group, leading to an eight coordinate DyO_8 environment that may be best described as a triangular dodecahedron (**Table S3** and **Figure S3a**), as determined by using the SHAPE[8] software. The molecular structure of $\mathbf{Dy_{(BTA)}}$ is similar to that of $\mathbf{Dy_{(TTA)}}$, where the two chelate TTA^{-1} ligands have been replaced by two chelate BTA^{-1} ligands. Similar to $\mathbf{Dy_{(TTA)}}$, SHAPE[8] analysis of the $\mathbf{Dy_{(BTA)}}$ suggests a triangular dodecahedron Dy^{III} coordination polyhedron (**Table S4** and **Figure S3b**). All Dy^{III}-O bond distances and O-Dy^{III}-O angles are typical for 8-coordinate Dy^{III} complexes (**Table S5**).[13] At the supramolecular level, neighbouring mononuclear molecules interact through weak H-bonds (C-H···O and C-H···F) forming 2-D supramolecular networks (**Figures S4-S5**). All geometrical parameters (Å, °) of the hydrogen-bonding motifs for $\mathbf{Dy_{(TTA)}}$ and $\mathbf{Dy_{(BTA)}}$ are given in **Table S6**.

Table S1. Crystallographic data and refinement details for $\mathbf{Dy_{(TTA)}}$ and $\mathbf{Dy_{(BTA)}}$.

Compound reference	Dy(TTA)	Dy(BTA)
Chemical formula	$C_{52}H_{38}DyF_6NO_9P_2S_2$	$C_{56}H_{42}DyF_6NO_9P_2$
Formula mass	1223.39	1211.34
Crystal system	Triclinic	Triclinic
a/Å	10.9357(5)	11.5109 (10)
b/Å	11.6426(6)	11.5374(10)
c/Å	12.3871(6)	12.3703(10)
α/°	102.425(2)	65.399(3)
β/°	102.326(2)	79.812(4)
γ/°	117.647(3)	62.339(3)
Unit cell volume/Å^3	1271.70(11)	1322.7 (2)
Temperature/K	100(2)	100(2)
Space group	*P*1	*P*1
No. of formula units/unit cell, *Z*	1	1
Radiation type	Mo Kα	Mo Kα
Absorption coefficient, μ/mm^{-1}	1.693	1.551
No. of reflections measured	11893	107267
No. of independent reflections	11893	11463
Data/restraints/parameters	11893/285/697	11463/383/677
Final R_1 values (all data)	0.0275	0.0251
Final $wR_2(F^2)$ values (all data)	0.0781	0.0626
Final R_1 values ($I > 2\sigma(I)$)	0.0275	0.0251
Final $wR_2(F^2)$ values ($I > 2\sigma(I)$)	0.0781	0.0626
Goodness of fit on F^2	1.034	1.060
Largest diff. peak and hole (eÅ^{-3})	3.318/-1.747	0.954/-0.853
CCDC number	2388654	2388655

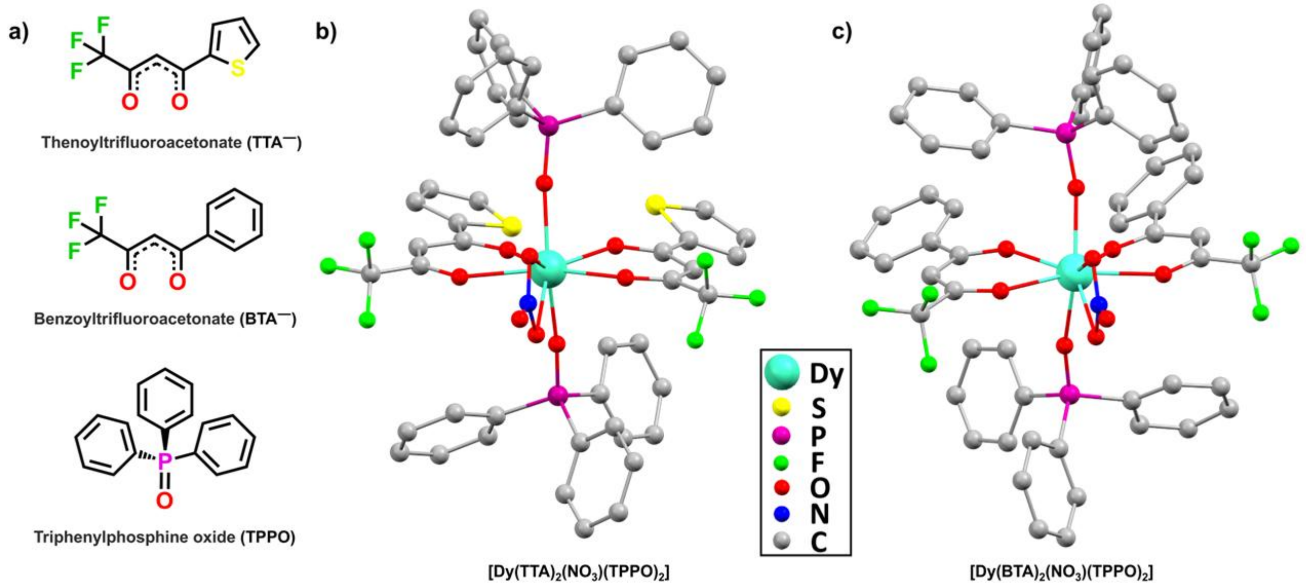

Figure S1. (a) Representation of 4,4,4-trifluoro-1-(2-thienyl)-1,3-butanedionate (TTA⁻), 4,4,4-trifluoro-1-phenylbutane-1,3-dionate (BTA⁻) and triphenylphosphine oxide (TPPO) ligands. Molecular structures of (b) **[Dy(TTA)$_2$(NO$_3$)(TPPO)$_2$] (Dy$_{(TTA)}$)** and (c) **[Dy(BTA)$_2$(NO$_3$)(TPPO)$_2$] (Dy$_{(BTA)}$)**.

Table S2. Cell parameters for **Dy$_{(TTA)}$** from the XRD data collections at 100 K, 296 K and 15 K.

Temperature/K	a/Å	*b*/Å	*c*/Å	*α*/°	*β*/°	*γ*/°	V/Å^3	Space group
**15(2)**	10.8893(9)	11.6099(13)	12.3711(9)	102.436(8)	102.327(7)	117.517(9)	1263.00(2)	*P*1
**100(2)**	10.9357(5)	11.6426(6)	12.3871(6)	102.425(2)	102.326(2)	117.647(3)	1271.70(11)	*P*1
**296(2)**	11.0770(5)	11.7661(5)	12.4855(5)	102.633(2)	102.100(2)	117.942(10)	1307.60(10)	*P*1

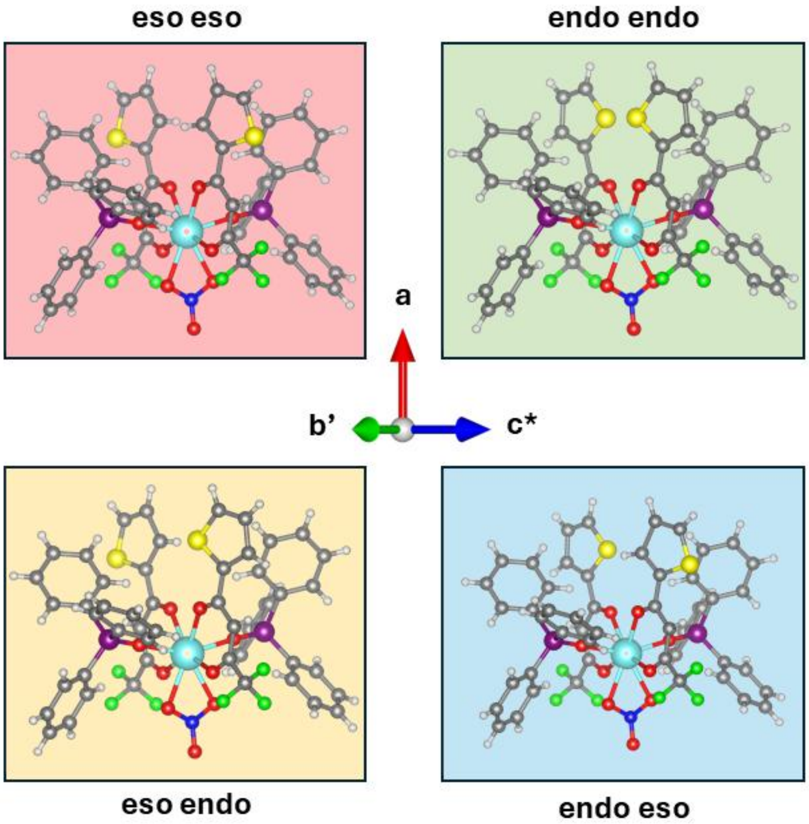

Figure S2. Representation of the four isomers generated by the molecular disorder localized at the level of the thiophene rings in **Dy$_{(TTA)}$**. The thiophene rings of both TTA^{-1} ligands were found to be disordered by an approximate 180° rotation around the C-C bond linking the rings to the β-diketonate skeleton.

Table S3. Results from Shape analysis for **Dy$_{(TTA)}$**.

Code	Label	Shape	Symmetry	CShM
1	OP-8	Octagon	D_{8h}	30.40887
2	HPY-8	Heptagonal pyramid	C_{7v}	22.94080
3	HBPY-8	Hexagonal bipyramid	D_{6h}	14.54462
4	CU-8	Cube	O_h	13.41382
5	SAPR-8	Square antiprism	D_{4d}	3.47405
**6**	**TDD-8**	**Triangular dodecahedron**	$\mathbf{D_{2d}}$	**1.10827**

7	JGBF-8	Johnson - Gyrobifastigium (J26)	D_{2d}	11.67701
8	JETBPY-8	Johnson - Elongated triangular bipyramid (J14)	D_{3h}	28.96650
9	JBTP-8	Johnson - Biaugmentedtrigonal prism (J50)	C_{2v}	3.15690
10	BTPR-8	Biaugmentedtrigonal prism	C_{2v}	2.79823
11	JSD-8	Snub disphenoid (J84)	D_{2d}	2.21651
12	TT-8	Triakis tetrahedron	T_d	14.09569
13	ETBPY-8	Elongated trigonalbipyramid	D_{3h}	25.30392

Table S4. Results from Shape analysis for **Dy$_{\mathbf{(BTA)}}$**.

Code	Label	Shape	Symmetry	CShM
1	OP-8	Octagon	D_{8h}	29.04245
2	HPY-8	Heptagonal pyramid	C_{7v}	22.57205
3	HBPY-8	Hexagonal bipyramid	D_{6h}	14.00090
4	CU-8	Cube	O_h	12.54378
5	SAPR-8	Square antiprism	D_{4d}	2.68738
**6**	**TDD-8**	**Triangular dodecahedron**	$\mathbf{D_{2d}}$	**1.30155**
7	JGBF-8	Johnson - Gyrobifastigium (J26)	D_{2d}	11.88466
8	JETBPY-8	Johnson - Elongated triangular bipyramid (J14)	D_{3h}	28.05615
9	JBTP-8	Johnson - Biaugmentedtrigonal prism (J50)	C_{2v}	2.68246
10	BTPR-8	Biaugmentedtrigonal prism	C_{2v}	2.37201
11	JSD-8	Snub disphenoid (J84)	D_{2d}	2.24389
12	TT-8	Triakis tetrahedron	T_d	13.35702
13	ETBPY-8	Elongated trigonalbipyramid	D_{3h}	24.77878

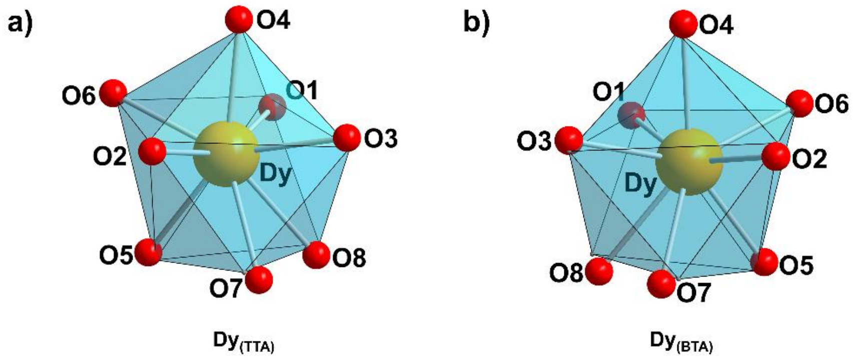

Figure S3. The triangular dodecahedron coordination polyhedrons of Dy^{III} centers in (a) **Dy$_{\mathbf{(TTA)}}$** and (b) **Dy$_{\mathbf{(BTA)}}$**. The smaller gray spheres define the vertices of the ideal triangular dodecahedrons.

Table S5. Selected interatomic distances (Å) and angles (˚) for **Dy$_{\mathbf{(TTA)}}$** and **Dy$_{\mathbf{(BTA)}}$**.

	Distance/angle	Symmetry code		Distance/angle	Symmetry code
		**Dy$_{\mathbf{(TTA)}}$**			
Dy-O(1)	2.240(4)	x, y, z	O(1)-Dy-O(4)	81.33(17)	x, y, z
Dy-O(2)	2.243(4)	x, y, z	O(2)-Dy-O(4)	79.87(16)	x, y, z
Dy-O(5)	2.338(4)	x, y, z	O(5)-Dy-O(4)	147.56(16)	x, y, z
Dy-O(6)	2.340(4)	x, y, z	O(6)-Dy-O(4)	75.20(15)	x, y, z
Dy-O(3)	2.343(4)	x, y, z	O(3)-Dy-O(4)	71.96(15)	x, y, z

Dy-O(4)	2.363(4)	x, y, z	O(1)-Dy-O(8)	74.99(17)	x, y, z
Dy-O(8)	2.438(4)	x, y, z	O(2)-Dy-O(8)	129.69(17)	x, y, z
Dy-O(7)	2.451(5)	x, y, z	O(5)-Dy-O(8)	73.39(16)	x, y, z
O(1)-Dy-O(2)	155.30(17)	x, y, z	O(6)-Dy-O(8)	134.52(16)	x, y, z
O(1)-Dy-O(5)	95.38(18)	x, y, z	O(3)-Dy-O(8)	72.04(15)	x, y, z
O(2)-Dy-O(5)	91.97(17)	x, y, z	O(4)-Dy-O(8)	134.95(16)	x, y, z
O(1)-Dy-O(6)	79.21(17)	x, y, z	O(1)-Dy-O(7)	127.20(18)	x, y, z
O(2)-Dy-O(6)	80.61(17)	x, y, z	O(2)-Dy-O(7)	77.47(17)	x, y, z
O(5)-Dy-O(6)	72.50(16)	x, y, z	O(5)-Dy-O(7)	71.35(16)	x, y, z
O(1)-Dy-O(3)	93.82(18)	x, y, z	O(6)-Dy-O(7)	136.61(16)	x, y, z
O(2)-Dy-O(3)	95.43(17)	x, y, z	O(3)-Dy-O(7)	72.45(16)	x, y, z
O(5)-Dy-O(3)	140.40(16)	x, y, z	O(4)-Dy-O(7)	135.30(16)	x, y, z
O(6)-Dy-O(3)	147.10(15)	x, y, z	O(8)-Dy-O(7)	52.22(17)	x, y, z
			**$Dy_{(BTA)}$**		
Dy-O(2)	2.231(4)	x, y, z	O(2)-Dy-O(6)	80.08(16)	x, y, z
Dy-O(1)	2.240(4)	x, y, z	O(1)-Dy-O(6)	80.22(15)	x, y, z
Dy-O(5)	2.324(3)	x, y, z	O(5)-Dy-O(6)	71.86(12)	x, y, z
Dy-O(3)	2.327(3)	x, y, z	O(3)-Dy-O(6)	146.68(12)	x, y, z
Dy-O(4)	2.360(3)	x, y, z	O(4)-Dy-O(6)	74.66(12)	x, y, z
Dy-O(6)	2.366(3)	x, y, z	O(2)-Dy-O(7)	75.51(16)	x, y, z
Dy-O(7)	2.439(4)	x, y, z	O(1)-Dy-O(7)	129.94(15)	x, y, z
Dy-O(8)	2.465(4)	x, y, z	O(5)-Dy-O(7)	73.40(13)	x, y, z
O(2)-Dy-O(1)	154.45(15)	x, y, z	O(3)-Dy-O(7)	72.77(13)	x, y, z
O(2)-Dy-O(5)	89.90(15)	x, y, z	O(4)-Dy-O(7)	132.37(13)	x, y, z
O(1)-Dy-O(5)	99.24(14)	x, y, z	O(6)-Dy-O(7)	137.13(13)	x, y, z
O(2)-Dy-O(3)	99.36(16)	x, y, z	O(2)-Dy-O(8)	127.16(17)	x, y, z
O(1)-Dy-O(3)	88.38(14)	x, y, z	O(1)-Dy-O(8)	78.38(16)	x, y, z
O(5)-Dy-O(3)	141.27(12)	x, y, z	O(5)-Dy-O(8)	72.09(13)	x, y, z
O(2)-Dy-O(4)	78.99(15)	x, y, z	O(3)-Dy-O(8)	72.43(13)	x, y, z
O(1)-Dy-O(4)	80.23(14)	x, y, z	O(4)-Dy-O(8)	139.16(13)	x, y, z
O(5)-Dy-O(4)	146.06(12)	x, y, z	O(6)-Dy-O(8)	134.02(13)	x, y, z
O(3)-Dy-O(4)	72.58(12)	x, y, z	O(7)-Dy-O(8)	51.92(15)	x, y, z

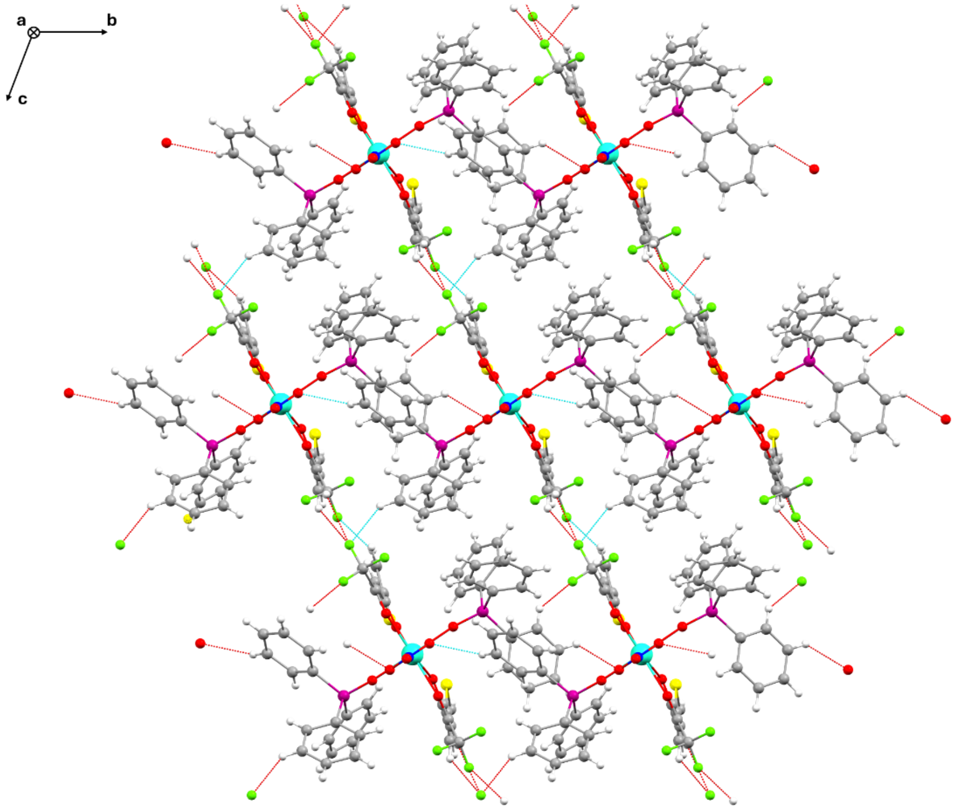

Figure S4. Part of the supramolecular arrangement of **Dy(TTA)** illustrating the 2D network formed by C−H···O and C−H···F hydrogen-bonding (red dashed lines).

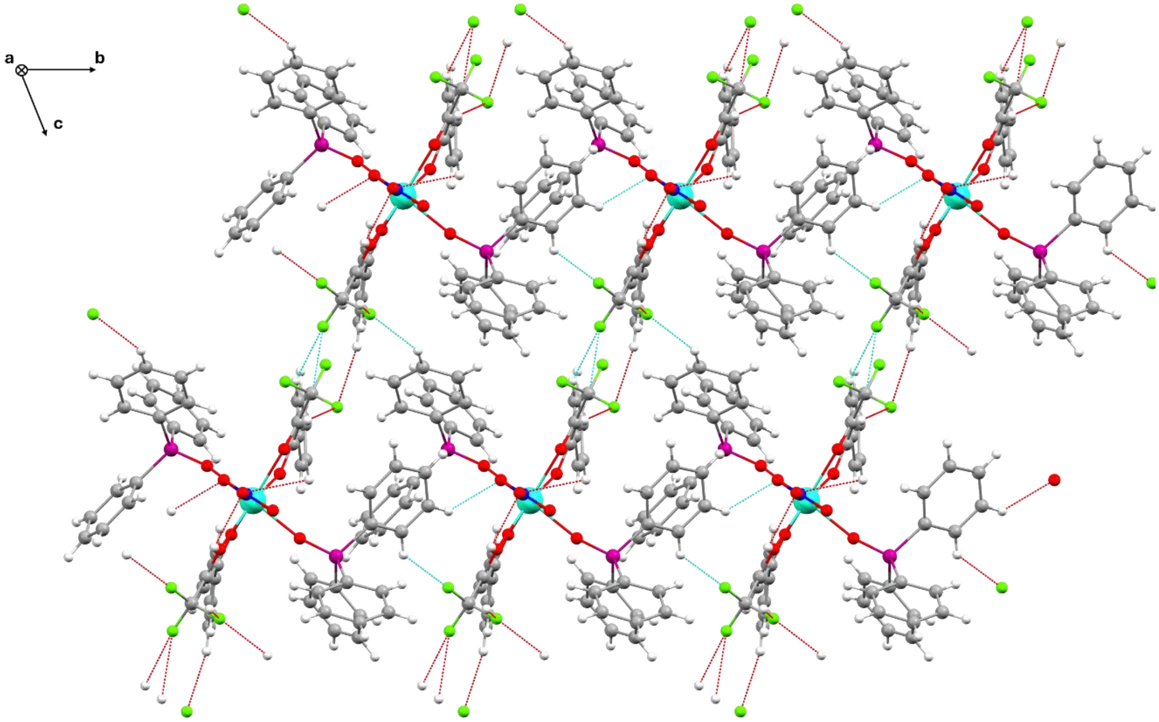

Figure S5. Part of the supramolecular arrangement of **Dy(BTA)** illustrating the 2D network formed by C–H···O and C–H···F hydrogen-bonding (red dashed lines).

Table S6. Geometry (Å, °) of the hydrogen bonds that are present in **Dy(TTA)** and **Dy(BTA)**.

D–H· · ·A	D–H	H· · ·A	D· · ·A	<(DHA)	Symmetry operation of A
			**Dy(TTA)**		
C9-H9···O7	0.95	2.46	3.237(11)	139	x, -1+y, z
C23-H23···O8	0.95	2.48	3.226(10)	136	1+x, 1+y, z
C24-H24···F1	0.95	2.47	3.224(10)	136	1+x, 1+y, z
C43-H43···F5	0.95	2.52	3.383(11)	152	x, -1+y, -1+z
			**Dy(BTA)**		
C11-H11···O7	0.95	2.49	3.273(9)	140	x, 1+y, z
C12-H12···F6	0.95	2.35	3.116(9)	137	x, 1+y, z
C54-H54···F4	0.95	2.53	3.461(15)	165	1+x, y, z
C55-H55···O9	0.95	2.48	3.320(11)	148	1+x, y, z

4. Powder X-ray Diffraction (PXRD)

The powder X-ray diffraction (PXRD) analysis was performed to confirm the phase purity of the **Dy$_{(TTA)}$** complex (**Figures S6**). The experimental PXRD pattern (blue) was recorded on a Bruker D8 Endeavor diffractometer with a CuKα (1.5418 Å) radiation source, equipped with LynxEye XE-T silicon strip detector, ranging from 5 to 35° (2θ), and was compared with the simulated pattern (grey) generated from the SCXRD data. The excellent match of the peaks' position and width between the experimental and simulated PXRD patterns confirms that the bulk material is phase-pure and corresponds to the single-crystal structure obtained by SCXRD. The slight differences in relative peak intensities are attributed to preferred orientation effects, which are common in powdered samples.

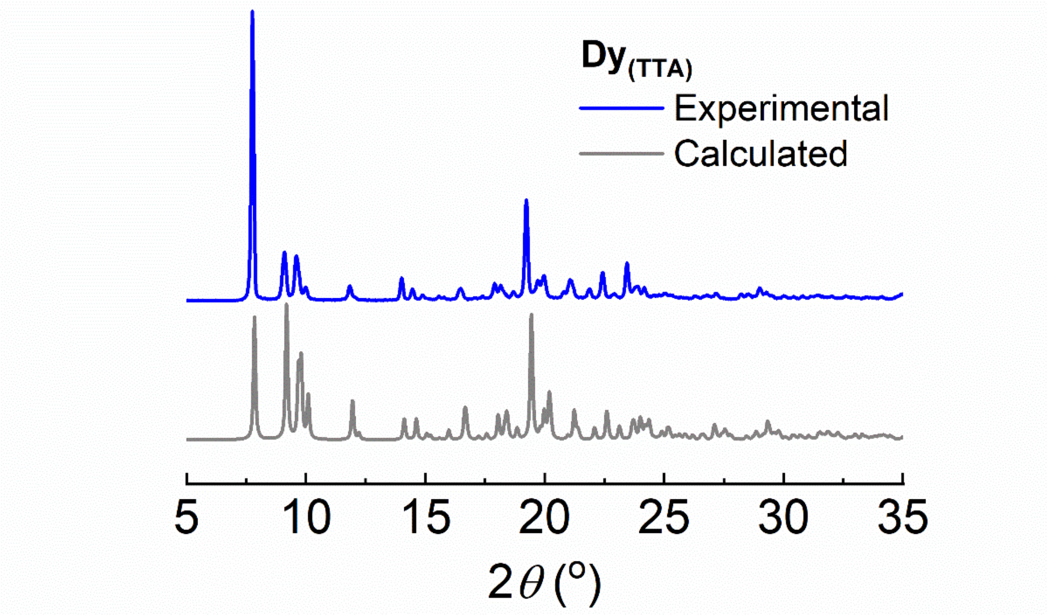

Figure S6. Simulated (grey) and experimental (blue) PXRD patterns for **Dy$_{(TTA)}$**.

5. Thermogravimetric analysis

The thermal analyses of both **Dy$_{(TTA)}$** and **Dy$_{(BTA)}$** complexes were performed using a Discovery TGA5500 thermal analysis system. Analytes were placed in a platinum pan and were heated to 900 °C with a ramp rate of 10 °C min^{-1}, using nitrogen as the carrier gas. The onset decomposition temperatures are 243 °C for **Dy$_{(TTA)}$** and 236 °C for **Dy$_{(BTA)}$**, respectively, highlighting their excellent thermal stability (**Figure S7**).

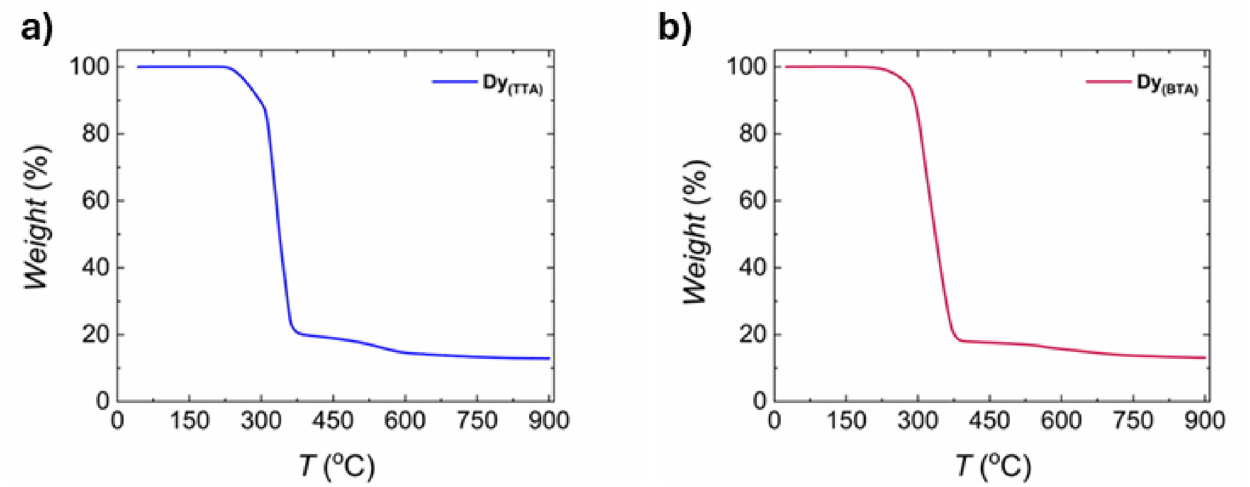

Figure S7. TGA analyses for a) $\mathbf{Dy_{(TTA)}}$ and b) $\mathbf{Dy_{(BTA)}}$.

6. Torque magnetometry

A small single crystal (mass equal to a few μg) was indexed and fixed with a known orientation (**Figure S8**) on a cantilever acting as the upper plate of a capacitor. The whole apparatus was placed in a static magnetic field and stabilized at a known temperature. Furthermore, the sample was rotated along an axis perpendicular to the applied magnetic field. The interaction between applied magnetic field and magnetic sample resulted in a τ force that induced a deflection of the cantilever. The correspondent change in capacitance was measured by varying the angle between cantilever (hence sample) and applied static magnetic field. These measurements were performed at different magnitudes of the applied magnetic field and in a selected temperature range providing information on the τ response as a function of the crystal orientation. Laboratory (*XYZ*) and orthogonalized crystal (*ab′c**) reference frames are related to the magnetic (*xyz*) reference frame by applying a suitable Euler rotation matrix, thus connecting the information between the τ response of the crystal and magnetic anisotropy associated with the single molecular complex unit.

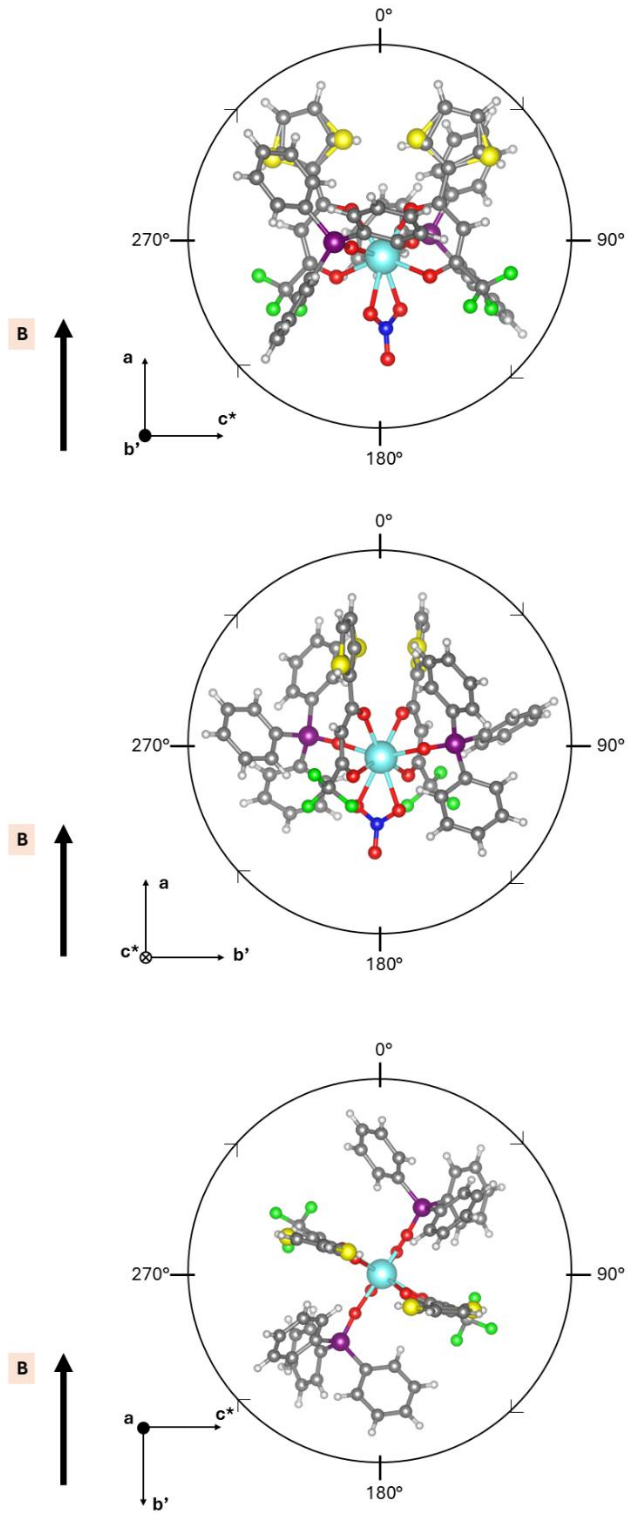

Figure S8. Orientation of the **Dy(TTA)** crystal.

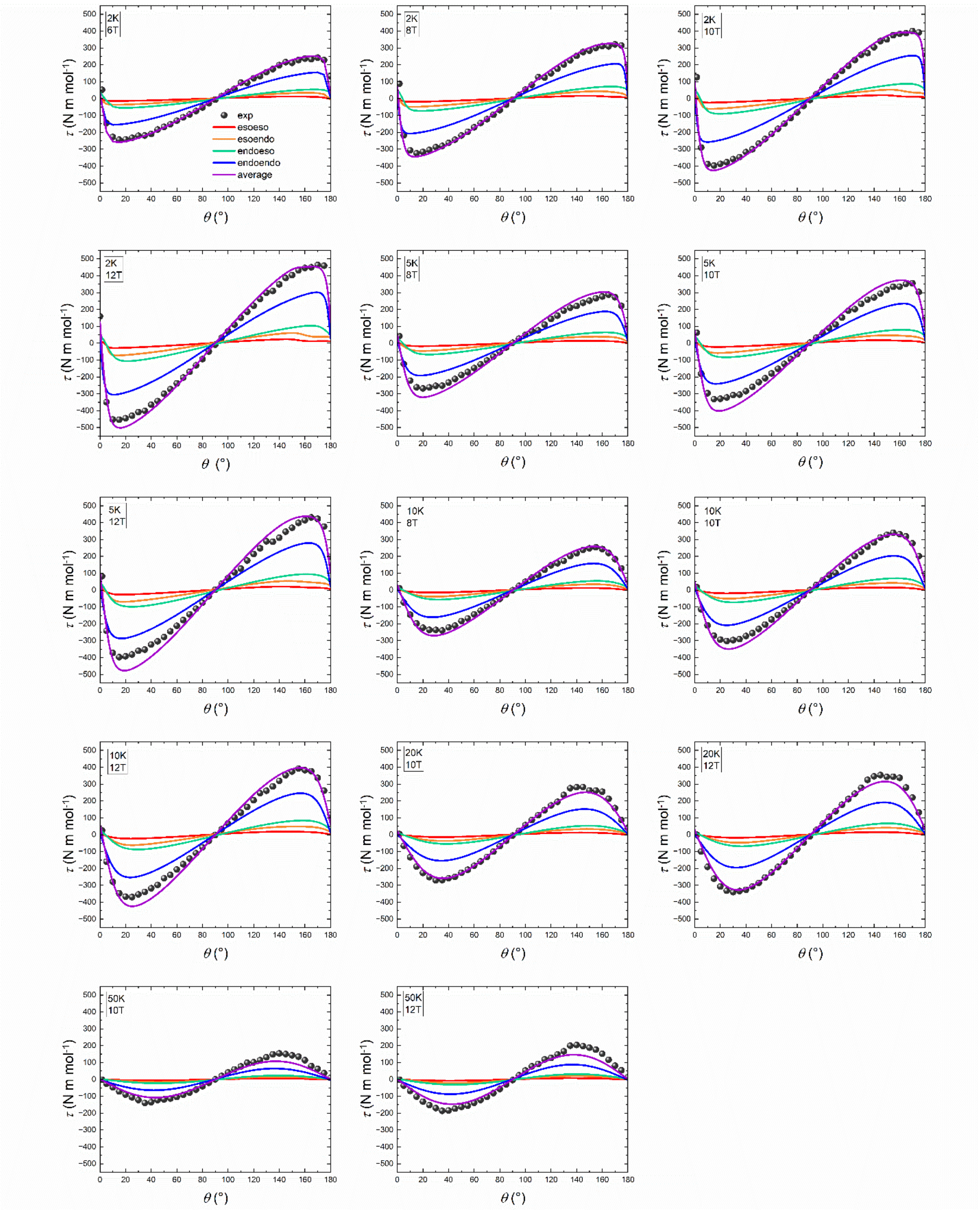

Figure S9. Angular dependence (rotation along *b′* axis as depicted in **Fig. S8**) of the magnetic torque (τ) measured on a single crystal of **Dy(TTA)** under an applied magnetic field between 6 - 12 T and in the temperature range 2 – 50 K. The dots correspond to the experimental data. The lines correspond to the contribution from each isomer due to molecular disorder (red, orange, green, and blue), and the weighted averaged resultant (violet).

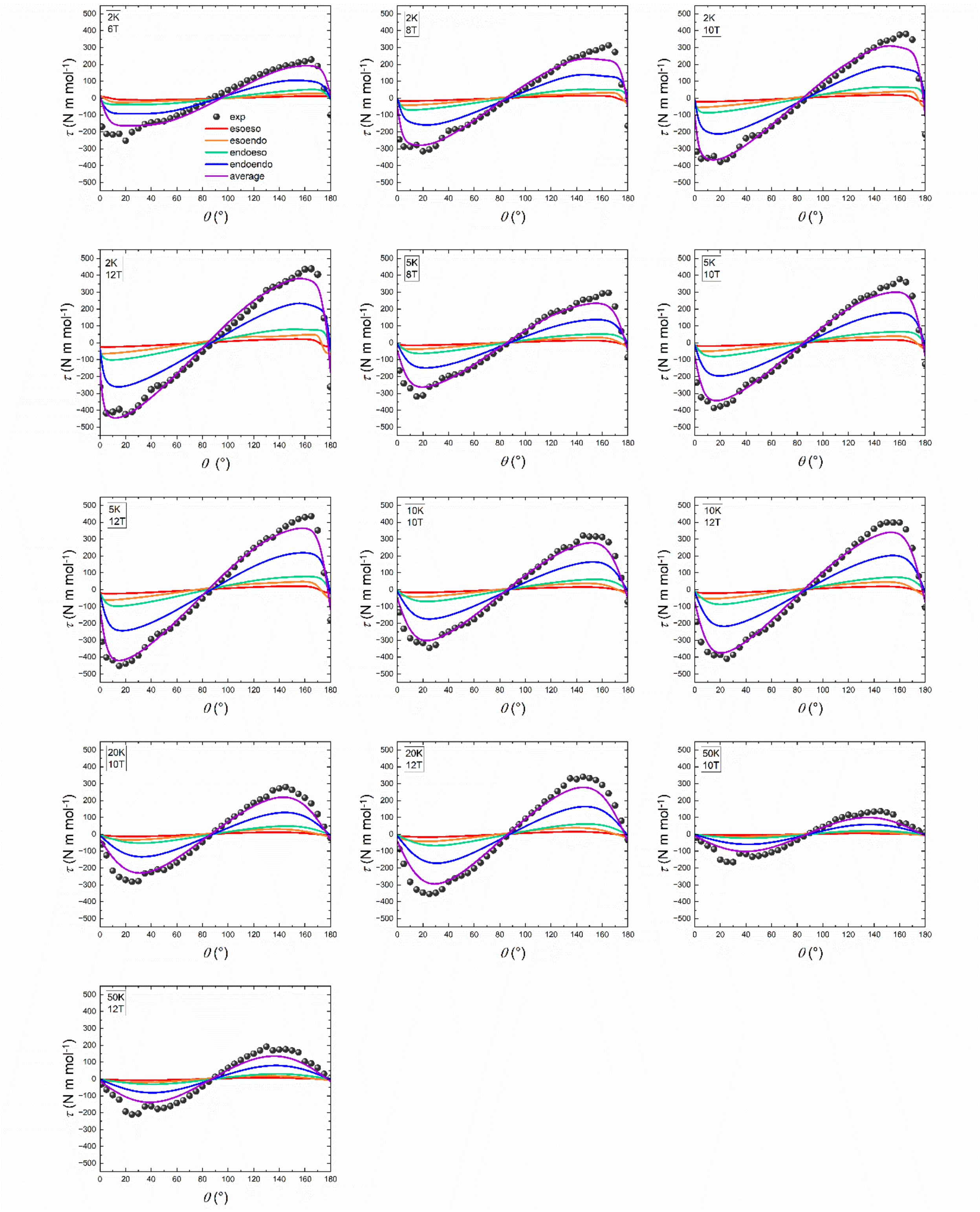

Figure S10. Angular dependence (rotation along *c** axis as depicted in **Fig. S8**) of the magnetic torque (τ) measured on a single crystal of **Dy$_{(TTA)}$** under an applied magnetic field between 6 - 12 T and in the temperature range 2 – 50 K. The dots correspond to the experimental data. The lines correspond to the contribution from each isomer due to molecular disorder (red, orange, green, and blue), and the weighted averaged resultant (violet).

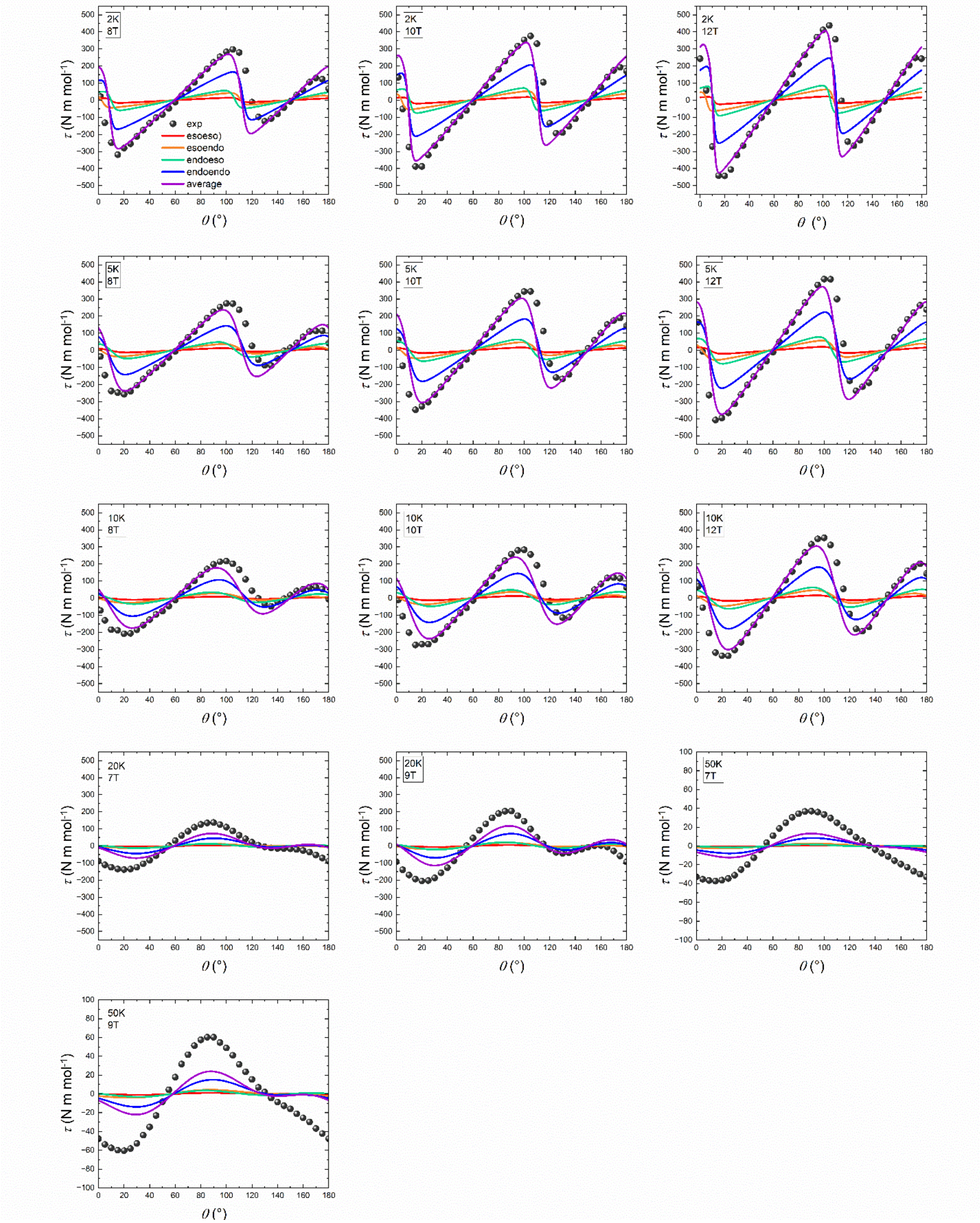

Figure S 11. Angular dependence (rotation along *a* axis as depicted in **Fig. S8**) of the magnetic torque (τ) measured on a single crystal of **Dy$_{(TTA)}$** under an applied magnetic field between 8 - 12 T and in the temperature range 2 – 50 K. The dots correspond to the experimental data. The lines correspond to the contribution from each isomer due to molecular disorder (red, orange, green, and blue), and the weighted averaged resultant (violet).

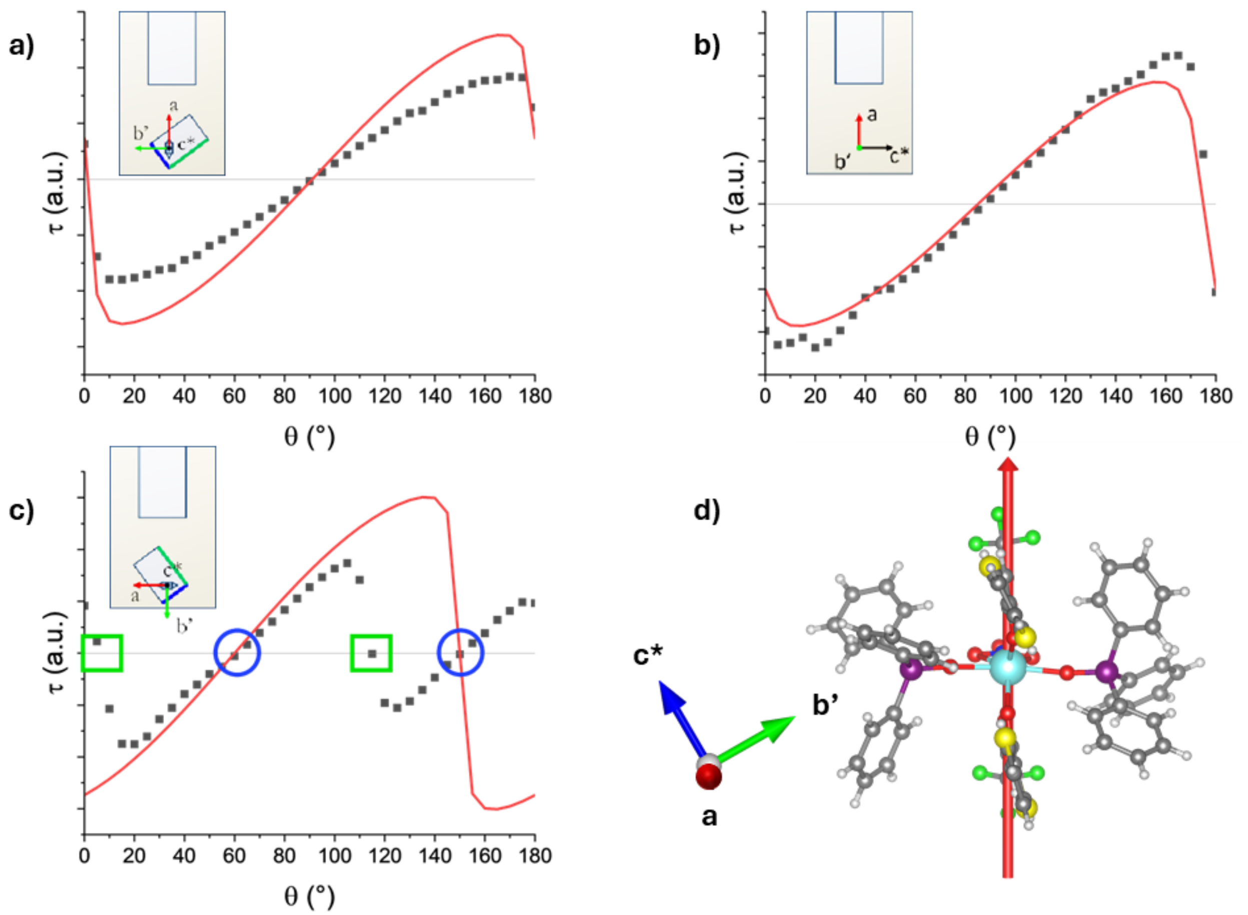

Figure S12. a, b, c) Angular dependence of the magnetic torque (τ) measured on a single crystal of **Dy(TTA)** under an applied magnetic field of 12 T at 2 K. The squares correspond to the experimental data. The lines correspond to simulated torque behavior by the axial (g_z = 20) one-spin 1/2 system model discussed in the main text and depicted in **d)**.

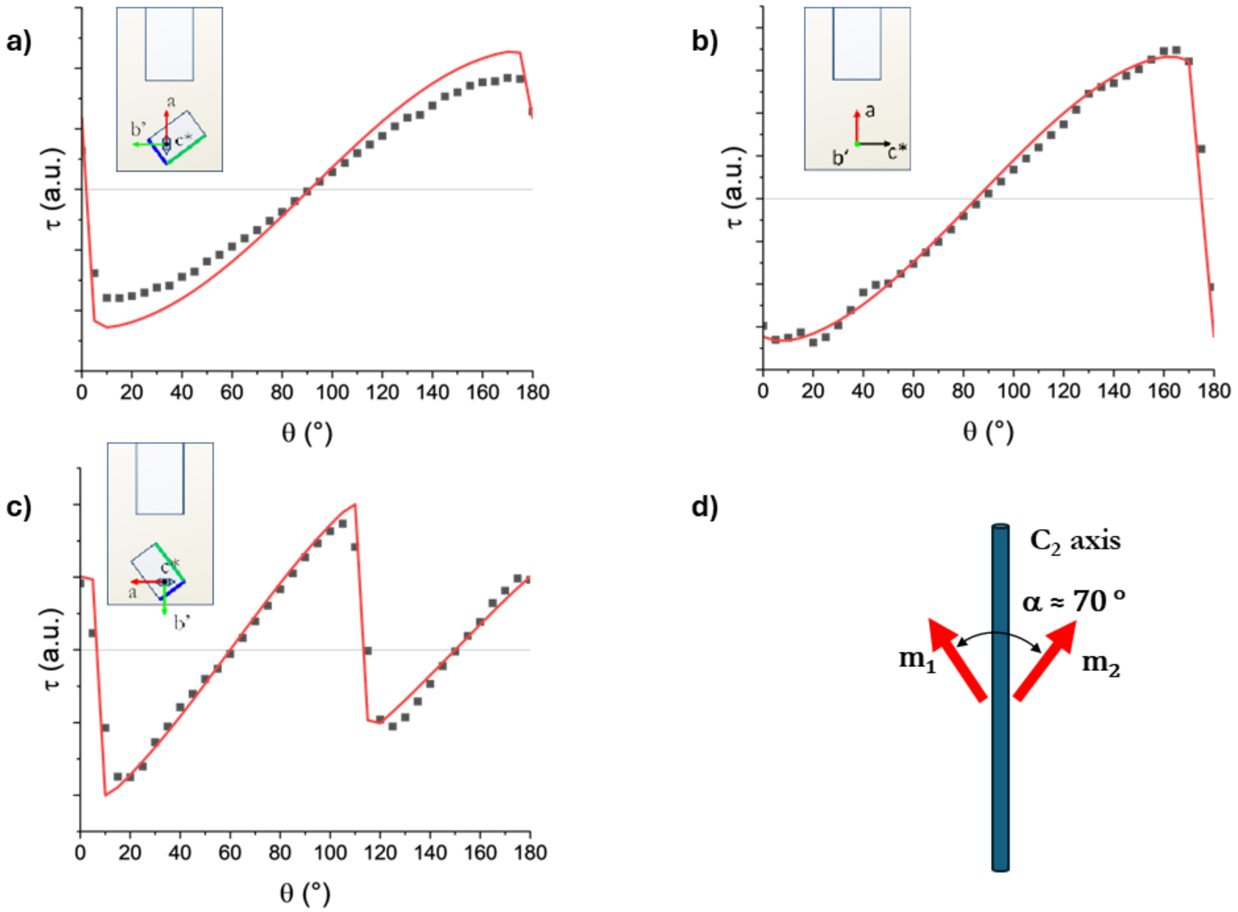

Figure S13. a, b, c) Angular dependence of the magnetic torque (τ) measured on a single crystal of **Dy(TTA)** under an applied magnetic field of 12 T at 2 K. The squares correspond to the experimental data. The lines correspond to simulated torque behavior by the axial (g_z = 20) two-spin 1/2 system model discussed in the main text and depicted in **d)**.

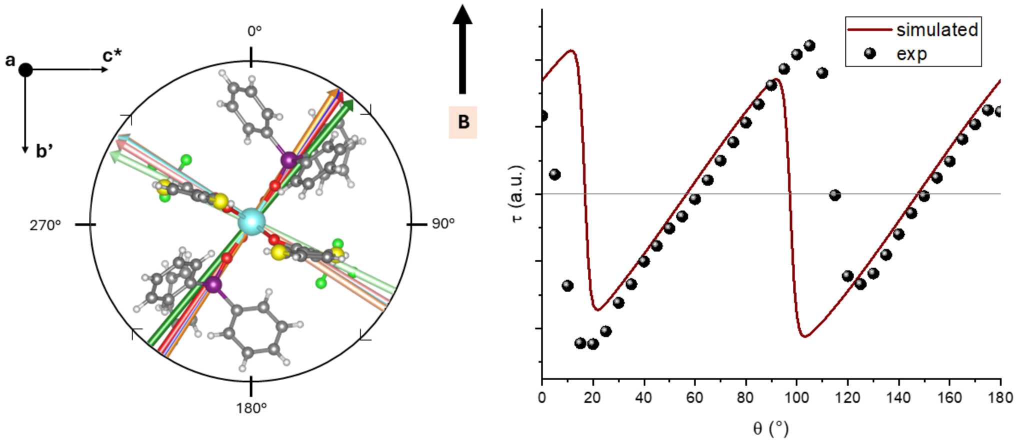

Figure S14. Angular dependence of the magnetic torque (τ) measured on a single-crystal of **Dy(TTA)** rotated along the a axis and under an applied magnetic field of 12 T at 2 K. The squares correspond to the experimental data. The lines correspond to simulated torque behavior by using the CF parameters extracted from the *ab initio* calculations. The

axes on the molecule represent the ground (strong colors) and first excited (light colors) doublet z axis. The four colors represent the four isomers.

$$\hat{H} = R^{-1}(\psi,\xi,\rho)\left(\sum_{k=2,4,6,8}\sum_{q=-k}^{k} B_k^q \hat{O}_k^q + g_J\mu_B \hat{J}\cdot\mathbf{B}\right)R(\psi,\xi,\rho)$$

Equation S1. For **Dy$_{TTA}$**, the angles ψ, ξ and ρ of the rotation matrix were obtained (see **Table S8**) by applying a fitting procedure that considered the four isomers generated by the molecular disorder. The contribution of each isomer was weighted according to its occupancy determined by single crystal X-ray analysis. The crystal field (CF) parameters extracted from *ab initio* calculations were multiplied by a scale factor equal to 1.5 (see main text).

Table S7. Euler angles (ψ, ξ and ρ, ZXZ extrinsic convention) obtained by fitting the experimental data with the CF parameters extracted from the *ab initio* calculations averaged from the molecular occupancy of each isomer and multiplied for a factor 1.5 (see main text).

	**EsoEso**	**EsoEndo**	**EndoEso**	**EndoEndo**
ψ	172.25	177.92	187.72	180.00
ξ	152.20	147.00	155.00	151.00
ρ	8.5300	22.505	16.180	18.000

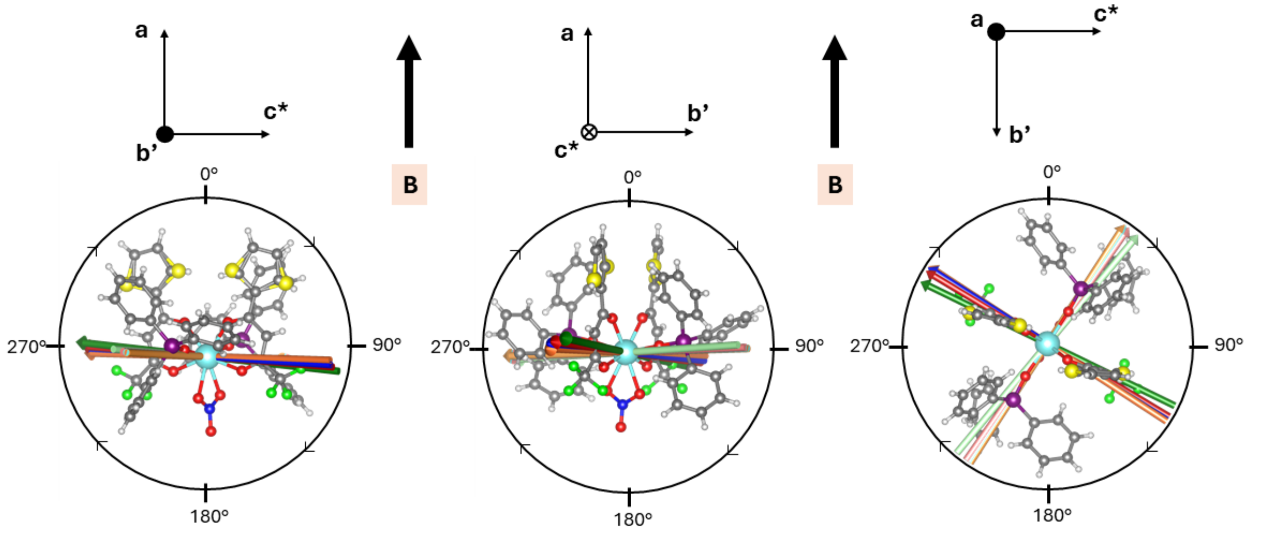

Figure S15. The three **Dy$_{(TTA)}$** crystal orientations studied by torque magnetometry. A representation of the anisotropy axes for the lowest m$_J$ doublets obtained from fitting the torque data is superimposed to the molecular structure.

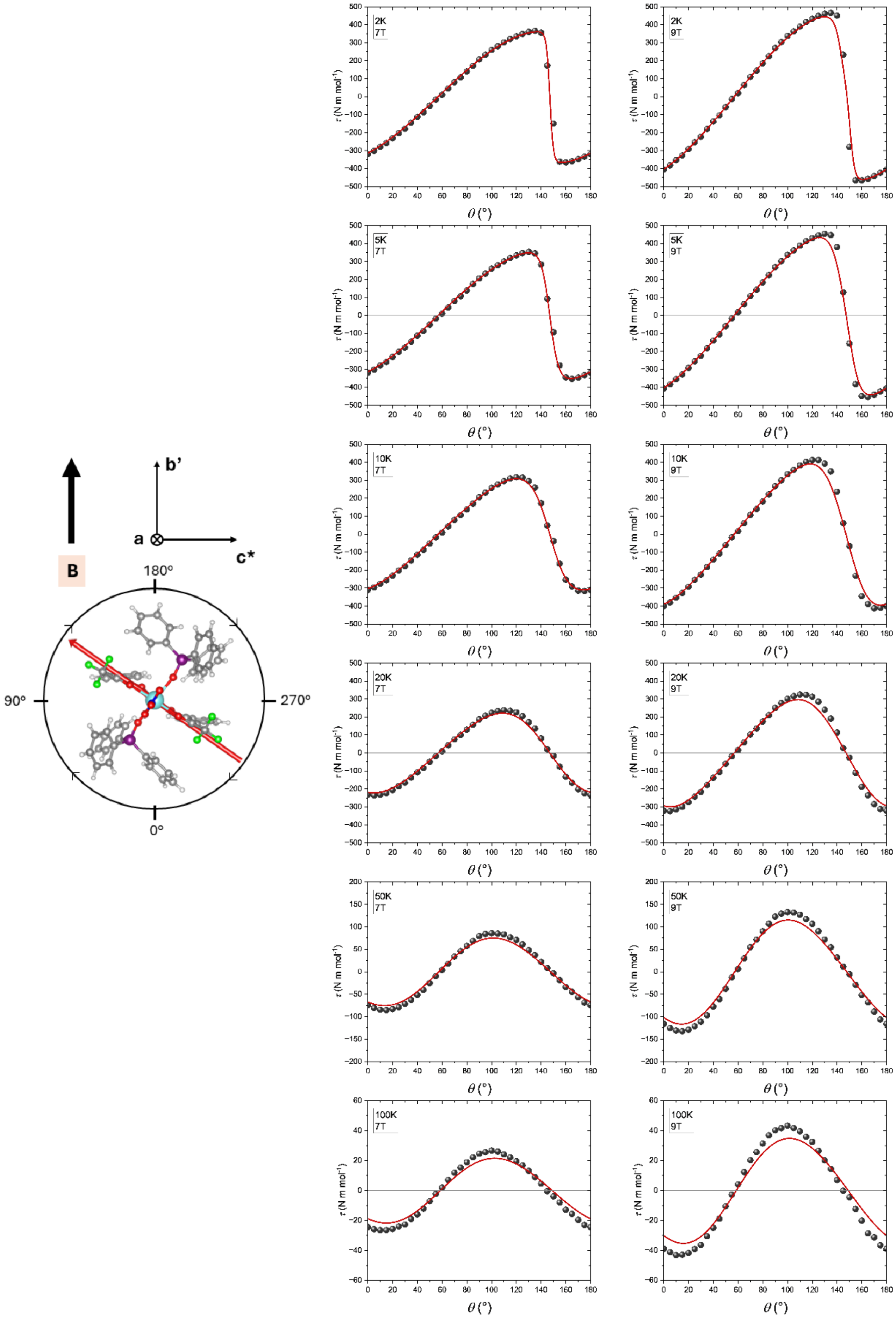

Figure S16. Angular dependence (rotation along the *a* axis as depicted) of the magnetic torque (τ) measured on a single crystal of **$Dy_{(BTA)}$** under an applied magnetic field of 7 and 9 T in the temperature range 2 – 100 K. The dots correspond to the experimental data. The lines correspond to the simulated torque behavior by using the CF parameters extracted from the ab initio calculations and multiplied for a factor 2.2 and the Euler angles in **Table S8**.

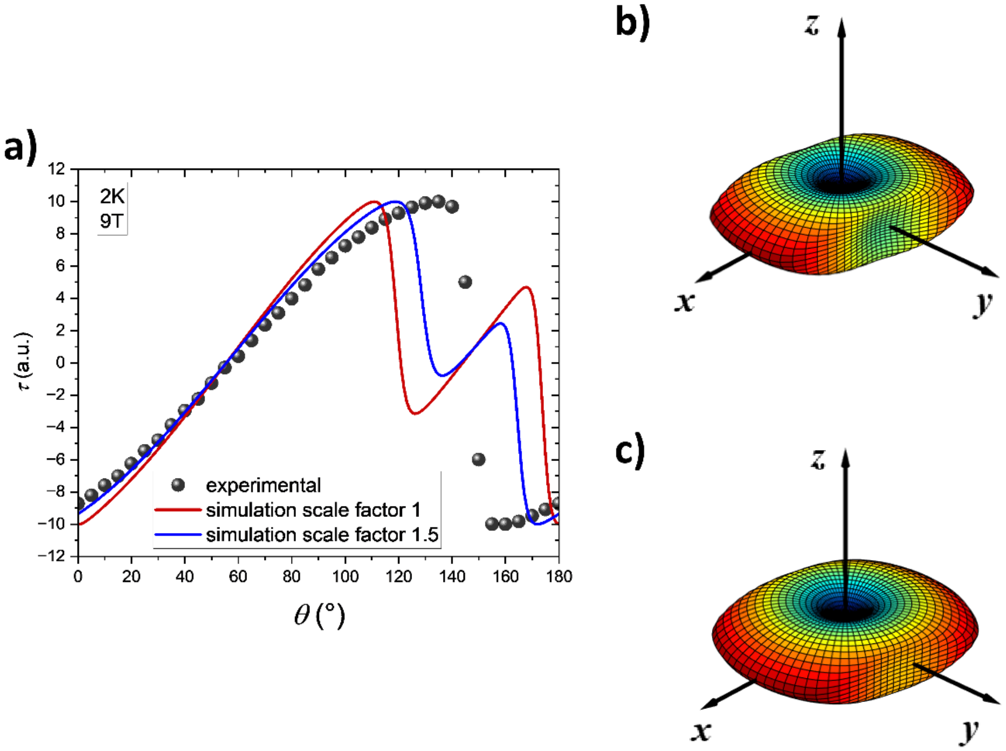

Figure S17. **a)** Angular dependence of the magnetic torque (τ) measured on a single crystal of **Dy$_{(BTA)}$** (rotation along the *a* axis as depicted in **Figure S16**) under an applied magnetic field of 9 T at 2 K. The dots correspond to the experimental data. The lines correspond to the simulated torque behavior by using the CF parameters extracted from the *ab initio* calculations without applying any scale factor (red) and applying a scale factor of 1.5 (blue). The different shapes of experimental and simulated curves (*i.e.* two and four zeros, respectively) are a clear indication of the significant energy gap underestimation of the first excited states by *ab initio* calculations (**Table S12**-**S13**). The free energy simulated at T = 2 K and B = 9 T and expressed in the magnetic reference frame xyz is depicted by using the CF parameters extracted from the *ab* initio calculations **b)** as is and **c)** multiplied for a scale factor equal to 1.5, corresponding to the red and blue lines in panel **a)**, respectively. It might be noticed that both the free energy representations exhibit two orthogonal easy axes (*i.e.* two minima are visible). However, increasing the scale factor (*i.e.* increasing the energy gap between the two lowest states), leads to a less pronounced minimum along the *y* axis.

Table S8. Euler angles (Ψ, ξ and ρ) used to simulate (see **Figure S16**) the magnetic torque (τ) behavior of **Dy$_{(BTA)}$**.

ψ	ξ	ρ
180	33	257

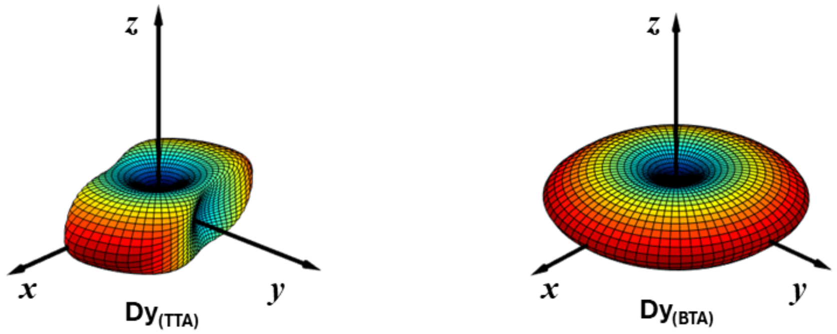

Figure S18. Comparison between the free energy of **Dy(TTA)** (CF parameters multiplied for a scale factor of 1.5) and **Dy(BTA)** (CF parameters multiplied for a scale factor of 2.2) simulated at T = 2 K and B = 7 T and expressed in the magnetic reference frame *xyz*. Only **Dy(TTA)** exhibits two orthogonal easy axes (*i.e.* two minima are visible).

7. Luminescence Spectroscopy

Excitation data were obtained using a Quanta Master 8075-21 spectrofluorometer (Horiba) equipped with a Hamamatsu R13456 PMT. An ozone-free PowerArc energy 75-watt xenon lamp was used as the radiation source. The excitation spectra were corrected in real-time according to the lamp intensity and the optical system of the excitation monochromator using a silicon diode as a reference. The sample was mounted inside a CS202*E-DMX-1AL closed-cycle helium cryostat system (Advanced Research Systems) controlled via a LakeShore 335 temperature controller. For the emission data, the sample was placed in an Oxford SpectromagPT cryogen-free magneto-optical superconducting magnet cryostat. Excitation was achieved using a Thorlabs SOLIS-365C LED (λ = 365 nm) followed by a OD4 400 nm shortpass filter (Edmund Optics). The excitation light was directed onto the sample through a series of lenses and then collected at a 180° angle using another set of lenses. The collected light was subsequently filtered through an OD4 400 nm longpass filter (Edmund Optics) and focused on an optical fiber with a plano-convex lens (Thorlabs LA4052 – ML). The optical fiber is connected to a Shamrock SR-750 spectrograph (1200 l/mm, 500 nm blaze grating) with an Andor iDus 420 CCD. A 10 µm slit was used for the luminescence data collection. Under these conditions, a peak resolution of 0.05 nm can be achieved. At the the $^4F_{9/2} \rightarrow {}^6H_{15/2}$ transition band (around 475 nm), it represents a peak resolution of approximately 2.2 cm^{-1}.

The Raman spectrum was obtained at 293 K with a MicroRaman Spectrometer (XploRA™ PLUS) with a 785 nm laser and a 1200 gr/mm grating.

Excitation and emission spectra are shown in **Figure S19**. In the excitation spectrum, a broad band is observed which arises from the excitation via the TPPO and TTA^{-1} ligands, revealing an efficient sensitization (antenna effect).[14] Additionally, sharp components are observed due to the direct Dy^{III} excitation. The emission spectrum obtained at 1.8 K reveals the characteristic transition bands from Dy^{III} ion arising from the $^4F_{9/2}$ emitter excited level to the $^6H_{15\text{-}9/2}$ levels.

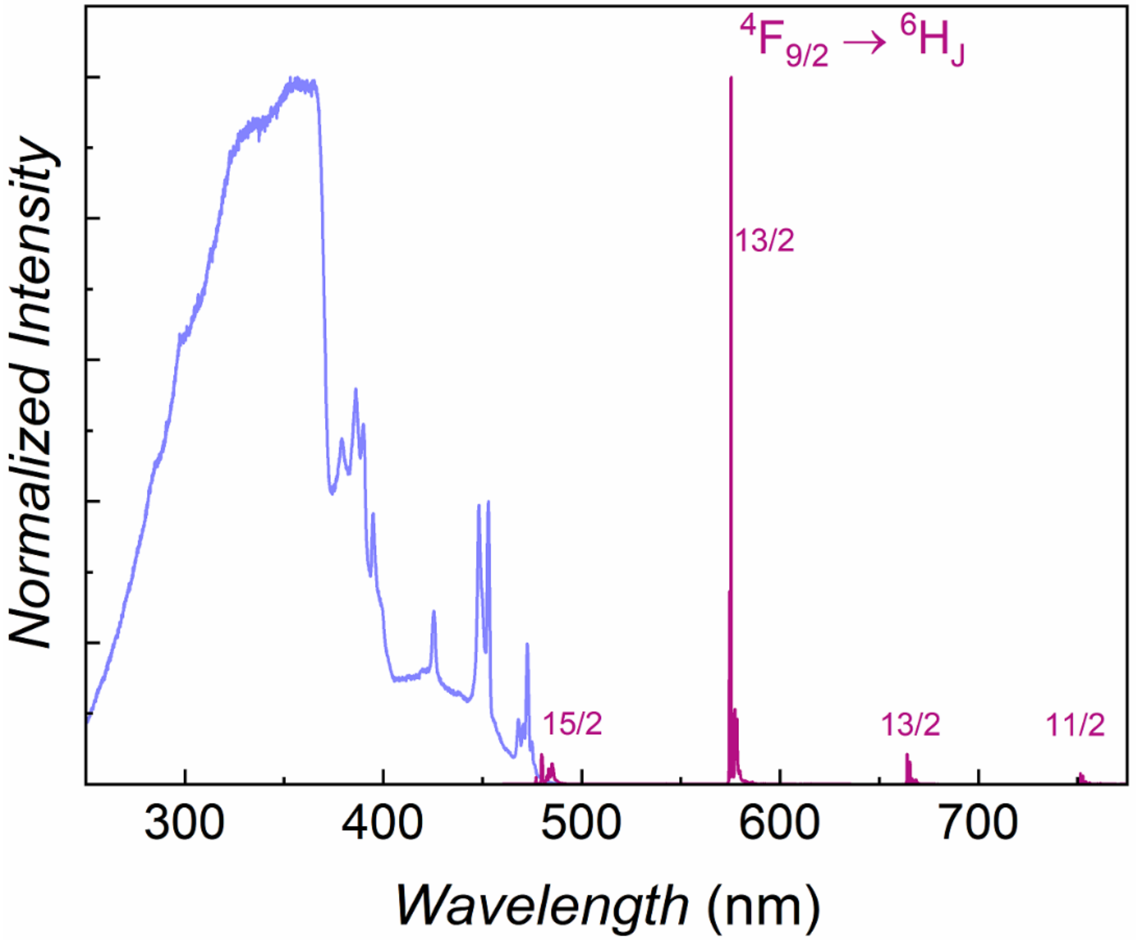

Figure S19. Excitation (violet line, λ_{em} = 575 nm, 10 K) and emission (purple line, λ_{ex} = 365 nm, 1.8 K) spectra for **$Dy_{(TTA)}$**.

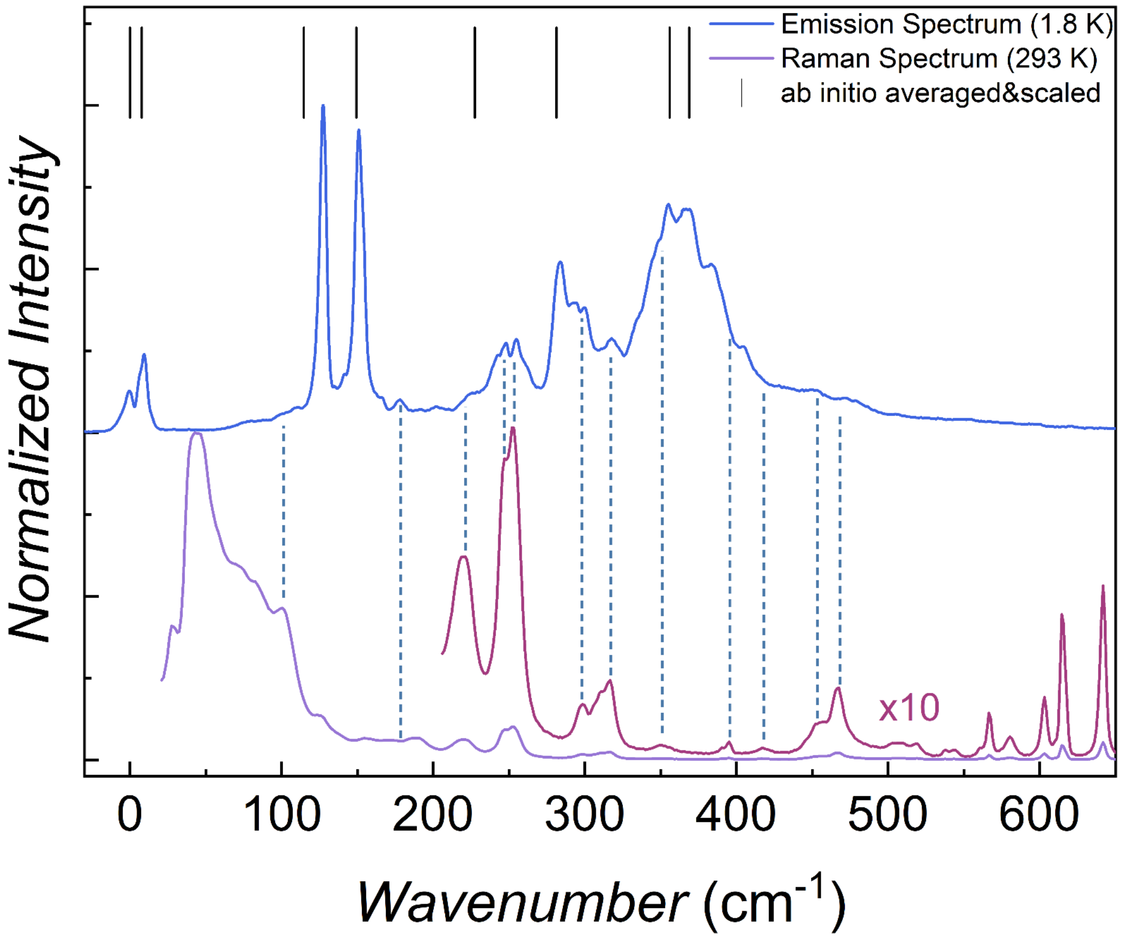

Figure S20. Emission (blue line, λ_{ex} = 365 nm, 1.8 K) and Raman (violet line, 293 K) spectra for **$Dy_{(TTA)}$**. The wine line is a 10x magnification of the Raman spectrum for the 200 – 650 cm^{-1} range. For the emission spectrum, we rescaled the x-axis by assuming the transition to the first CF sublevel as the 0 cm^{-1}.

Table S9. The calculated (and weighted average for the disordered molecular occupancy) energy splitting of the $^6H_{15/2}$ level as is and multiplied for a scale factor equal to 1.5.

**ΔE(*Ab initio*, average) / cm^{-1}**	**ΔE(*Ab initio*, average) * 1.5 / cm^{-1}**
0	0
5.2252878	7.8379317
76.5038136	114.7557204
99.6082361	149.4123542
151.6518828	227.4778242
187.5678793	281.351819
237.345191	356.0177865
245.9055452	368.8583178

8. Dc and ac Magnetic Studies

Magnetic susceptibility measurements for **$Dy_{(TTA)}$** were obtained using a Quantum Design MPMS3 SQUID magnetometer. The sample was prepared by restraining in silicon grease the crushed crystalline compound (18.8 mg) and sealing it in a polyethylene membrane, for which diamagnetic corrections were applied. The sample was subjected to direct current (dc) fields up to 7 T, while alternating current (ac) measurements took place both in the absence and presence of a static field of 0.1 T and a frequency of 1000 Hz. The dc magnetic susceptibility was measured between 300 and 1.8 K under an applied field of 0.1 T (**Figures S21**). At room temperature the χT product of 14.14 cm^3 K mol^{-1} for **$Dy_{(TTA)}$** is in good agreement with the expected value of one Dy^{III} ion (S = 5/2, L = 5, $^6H_{15/2}$, g = 4/3, C = 14.17 cm^3 K mol^{-1}). Upon lowering of the temperature, the χT product starts to gradually decrease reaching a minimum of 9.92 cm^3 K mol^{-1} for **$Dy_{(TTA)}$** at 1.8 K. This decrease can be attributed to thermal depopulation of the Stark sublevels, as well as the presence of magnetic anisotropy (MA). To further confirm this, the field dependence of the magnetization was measured between 1.9 and 7 K (**Figures S22**). No saturation of the magnetization is observed for the *M vs. H* plot at 1.9 K, even at fields as high as 7 T, indicating the presence of low-lying excited states and MA. Following this, the ac susceptibility was probed. The in-phase (χ') and out-of-phase (χ'') ac susceptibilities were measured at a frequency of 1000 Hz with different temperatures both in the absence and presence of a static H_{dc} = 0.1 T (**Figure S23**). Under zero field, no peak of the ac susceptibility was observed, while when H_{dc} = 0.1 T, only a tail of the χ'' susceptibility was observed in the low temperature region, signifying that quantum tunneling of the magnetization (QTM) dominates the relaxation of the magnetization.

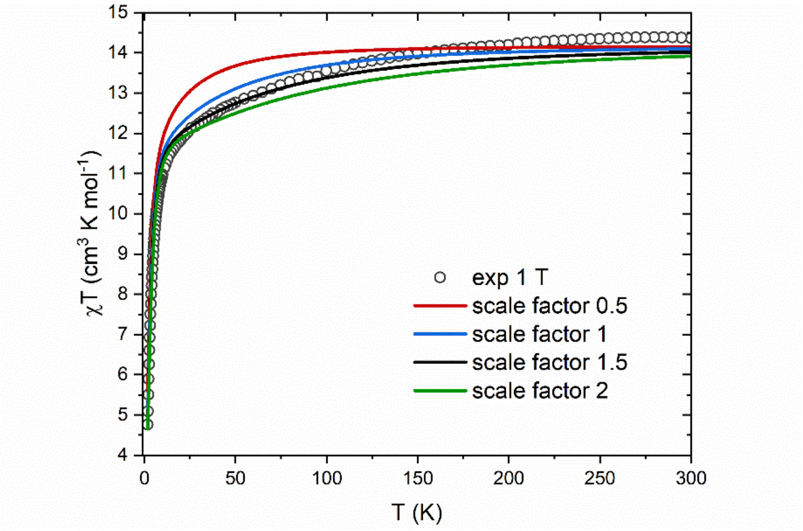

Figure S21. Experimental variable temperature χT plot of **Dy(TTA)** under an applied static field of 1 T (circles). Simulations of the χT vs. T plot using CF set multiplied for a scale factor equal to 0.5 (red line), 1 (blue line), 1.5 (black line), and 2 (green line).

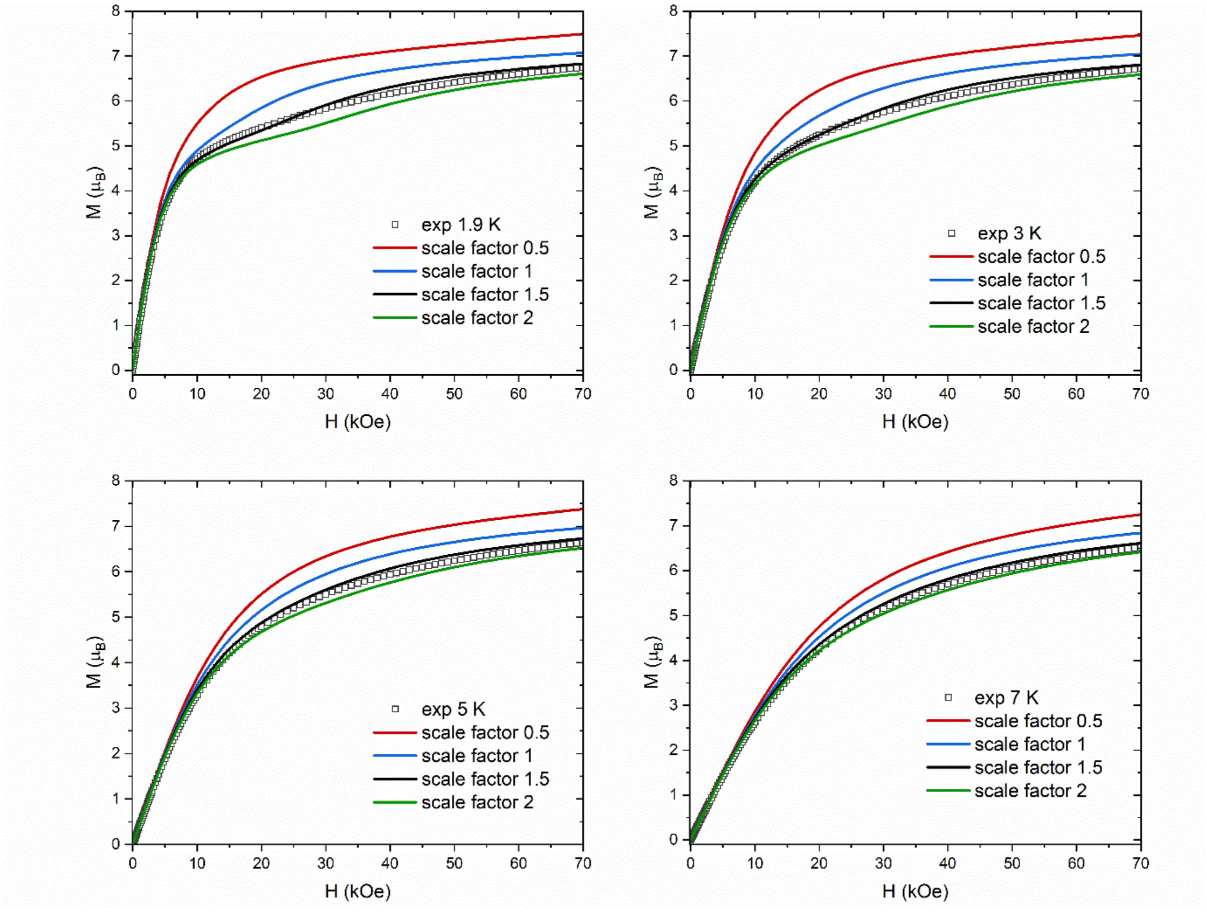

Figure S22. Experimental field dependence of the magnetization of **Dy(TTA)** at 1.9, 3, 5 and 7 K between 0 and 70 kOe (squares). Simulations of the M vs. H plot using CF set multiplied for a scale factor equal to 0.5 (red line), 1 (blue line), 1.5 (black line), and 2 (green line).

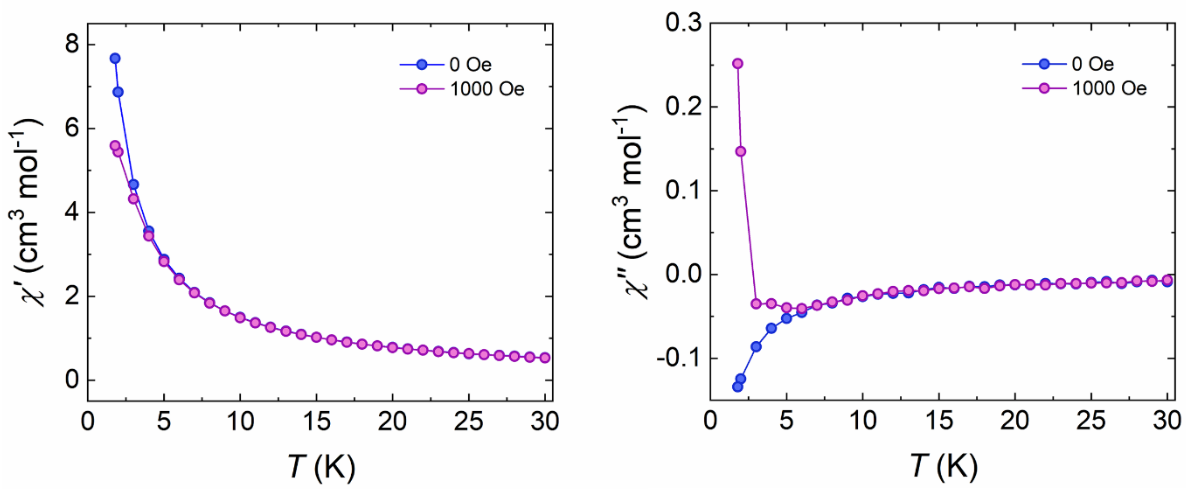

Figure S23. Ac susceptibility measurements for **Dy(TTA)**, performed at variable temperatures and 1000 Hz in the absence and presence of an applied dc field of 1000 Oe.

9. Complete Active Space Self-Consistent Field (CASSCF) Ab Initio Calculations

The calculations were performed on X-ray crystal structures without further geometry optimizations. All the simulations were performed with ORCA 5.0.2 Quantum Chemistry Software Package.[15] Second-order Douglas-Kroll-Hess Hamiltonian has been always employed to consider scalar relativistic corrections. SARC2-QZVP basis sets[16] were employed for the lanthanide ion, while DKH-def2-TZVP[17] basis sets were used for all other atomic species. Resolution of identity (RI) approximation was applied[18] with the default settings for the integration grids. AUTOAUX feature was used to automatically generate the corresponding auxiliary basis sets. The energy ladder of the electronic states for every lanthanide ion have been computed within the CASSCF method followed by Spin Orbit coupling calculations within the Quasi-Degenerate Perturbation Theory (QDPT) and mean field approaches. The chosen active space for the lanthanides consisted of the nine unpaired electrons in the seven 4f -orbitals of the dysprosium ion in the oxidation state +3: CAS (9,7). 21 sextuplets and 224 quartets were computed and included in the following spin-orbit calculation. The g-tensor and the Extended Stevens' Operator (ESO) were calculated with the SINGLE_ANISO module[19,20] as implemented in ORCA.

Table S10. Energy levels' splitting of the 8 Kramers' doublets belonging to the fundamental $^6H_{15/2}$ multiplet for the four isomers generated by molecular disorder *for* **Dy(TTA)**. All energies are reported in cm^{-1}.

Kramers'Doublet	Endo Endo	Eso Eso	Eso Endo	Endo Eso
E₀	0.00000000	0.00000000	0.00000000	0.00000000
E₁	5.90373608	3.89582813	6.66673960	2.84935142
E₂	78.32369240	71.99244143	77.29540984	72.66341948
E₃	100.30460340	97.77509191	99.48940879	98.76121344
E₄	152.71284092	149.01838039	151.51666074	150.29111379

E_5	188.81740491	184.50551876	187.34372150	186.03863674
E_6	237.79055724	236.91222007	238.02054506	236.91599511
E_7	246.35169240	245.58796389	246.32180025	245.65404791

Table S11. Computed g-tensors and directions of the main magnetic axes for the ground and first excited Kramers' doublets of the four isomers generated by molecular disorder for $\boldsymbol{Dy_{(TTA)}}$.

**Endo Endo**					
**Kramers' doublet**	**Component**	**Value**	**a**	**b'**	**c***
E_0	g_x	0.02007702	0.973781	-0.147636	-0.173072
	g_y	0.25299136	0.225698	0.531788	0.816248
	g_z	18.98028236	-0.028470	-0.833909	0.551166
E_1	g_x	0.20173210	0.487426	-0.721187	0.492245
	g_y	0.69536591	0.871341	0.438156	-0.220871
	g_z	18.52202653	-0.056391	0.536572	0.841968

**Eso Eso**					
**Kramers' doublet**	**Component**	**Value**	**a**	**b'**	**c***
E_0	g_x	0.06851989	0.998549	-0.051686	-0.015084
	g_y	0.71385787	0.041968	0.571681	0.819402
	g_z	18.28027446	-0.033728	-0.818846	0.573021
E_1	g_x	0.09014172	0.699916	-0.585316	0.409296
	g_y	1.00177389	0.710631	0.628123	-0.316963
	g_z	17.74626190	-0.071565	0.512705	0.855577

**Eso Endo**					
**Kramers' doublet**	**Component**	**Value**	**a**	**b'**	**c***
E_0	g_x	0.01620857	0.979333	-0.130310	-0.154683
	g_y	0.24392495	0.200617	0.528683	0.824771
	g_z	19.06756669	-0.025697	-0.838757	0.543899
E_1	g_x	0.19168438	0.414950	-0.756629	0.505301
	g_y	0.76829787	0.909509	0.360027	-0.207784
	g_z	18.56920082	-0.024706	0.545796	0.837554

**Endo Eso**					
**Kramers' doublet**	**Component**	**Value**	**a**	**b'**	**c***
E_0	g_x	0.16064458	0.998730	-0.036798	0.034416
	g_y	1.15772434	-0.002957	0.639096	0.769122
	g_z	17.03787075	-0.050297	-0.768247	0.638175
E_1	g_x	0.16504875	-0.911526	0.320139	-0.258130
	g_y	1.29828427	-0.398835	-0.841204	0.365110
	g_z	16.55520492	-0.100254	0.435759	0.894463

Table S12. Extended Stevens' Operators (ESOs) B_k^q coefficients mapped on the fundamental $^6H_{15/2}$ multiplet for the four **Dy(TTA)** derivatives arising from molecular disorder. The quantization axis is the easy axis of the computed fundamental Kramers' doublet.

k	q	Endo Endo	Eso Eso	Eso Endo	Endo Eso
2	-2	0.29063952955296E+00	0.24741907771790E+00	0.19385497998897E+00	0.29963734611662E+00
2	-1	0.22911679378171E+00	0.13852245591690E+00	0.24007569595258E+00	0.50462616604048E-01
2	0	-0.45020942260138E-01	-0.11738842947973E-01	-0.37702377493233E-01	-0.20179523953100E-01
2	1	0.48812093239581E+00	0.55220681782157E+00	0.48469468318072E+00	0.61236007529754E+00
2	2	0.12028089193671E+00	0.15967293594343E+00	0.13741327152616E+00	0.22538648529476E+00
4	-4	0.16586328122717E-02	0.14401149135845E-02	0.17308178918000E-02	0.16186466793910E-02
4	-3	-0.57461580624926E-02	0.25810556449968E-02	-0.58333572143239E-02	0.14241771377381E-01
4	-2	-0.81416840339616E-02	-0.10070368914345E-02	-0.72873058164534E-02	0.22897077396301E-02
4	-1	-0.41041405336017E-03	0.58915687142868E-02	-0.19075359086035E-02	0.21407227510418E-01
4	0	-0.68698486821602E-02	-0.68232085153889E-02	-0.68904030443502E-02	-0.61779129697448E-02
4	1	-0.66457969725027E-02	-0.72634723604589E-02	-0.61309568413721E-02	-0.77832015200243E-02
4	2	-0.16886490591394E-01	-0.18511671495629E-01	-0.17233484225293E-01	-0.16484017081513E-01
4	3	-0.78707739104810E-02	-0.10781772374484E-01	-0.74259695905529E-02	-0.11165147341496E-01
4	4	0.21844212251413E-04	0.13802564765241E-02	0.27176308379361E-03	0.18088031402731E-02
6	-6	0.19802966922695E-04	0.93929203887067E-05	0.21195004021009E-04	-0.52671144035305E-05
6	-5	0.16539604096244E-03	0.56721859780863E-05	0.16297357401366E-03	-0.18964296748072E-03
6	-4	0.12674126904605E-03	0.29507453340134E-04	0.11609282785033E-03	0.12198824927203E-04
6	-3	-0.78789133586895E-04	-0.79182761067748E-05	-0.65307611136003E-04	-0.62492664080879E-05
6	-2	-0.47684532813969E-04	-0.13882952680252E-04	-0.43452799873374E-04	-0.76435341381702E-05
6	-1	0.30035118506900E-04	0.90845875281443E-05	0.27444850255164E-04	-0.33812750540335E-05
6	0	0.11478939846773E-04	0.11283284930575E-04	0.11600079090707E-04	0.10340970220477E-04
6	1	0.97633048909507E-04	0.10536620902745E-03	0.95809099267766E-04	0.11685969444638E-03
6	2	-0.84310408121442E-04	-0.92683084946515E-04	-0.88527046940801E-04	-0.84044630949251E-04
6	3	-0.12310467271171E-03	-0.15391238628212E-03	-0.12654542195850E-03	-0.17486017406561E-03
6	4	0.98015905797942E-04	0.15739063569265E-03	0.11190472690856E-03	0.14981322232608E-03
6	5	0.57656561868777E-04	0.18709048212778E-03	0.61326573643840E-04	0.21045114717136E-03
6	6	-0.23152282335975E-05	0.21338529011037E-04	0.75676052985012E-06	0.18759754366363E-04
8	-8	-0.45755878554664E-08	-0.11673168567394E-08	-0.46507203631776E-08	0.20066313784042E-08
8	-7	-0.24888835326016E-07	0.18408899070191E-08	-0.25103675929212E-07	0.42605485959163E-07
8	-6	-0.14907047984050E-07	-0.71984499294305E-09	-0.13276011288508E-07	-0.17526307584477E-08
8	-5	0.20826421199319E-06	0.26865846562327E-07	0.19749617412259E-06	-0.12772455803212E-06
8	-4	0.45161704522498E-07	0.66178453924295E-08	0.41711808319417E-07	-0.10220275864012E-07
8	-3	-0.11010745971139E-07	-0.50565320668926E-07	0.60962049754816E-08	-0.17268697490097E-06
8	-2	0.68795157904386E-07	0.17531490099045E-07	0.59457095054822E-07	0.22007211514923E-07
8	-1	-0.55610132397544E-08	-0.11035590274352E-07	-0.20821987050784E-08	-0.31278182338275E-07
8	0	0.32404852248024E-10	-0.26904448184753E-10	0.28319506062756E-10	-0.10334218307841E-08
8	1	-0.15329475870437E-07	-0.15937482533014E-07	-0.15435306213654E-07	-0.18646889382518E-07
8	2	0.13546577002449E-06	0.14467614415184E-06	0.13955772716084E-06	0.10325246812102E-06
8	3	0.17413285354273E-08	0.20127184278800E-08	-0.96122952216040E-08	-0.21554815874447E-08
8	4	0.28730769480794E-07	0.51360273908518E-07	0.34219237397836E-07	0.32878588663910E-07
8	5	0.69961108465848E-07	0.23337246231122E-06	0.82915081379300E-07	0.24371479883956E-06
8	6	-0.78601678465733E-08	-0.14573056890936E-07	-0.10067357651849E-07	-0.13449780358359E-07
8	7	0.18767376401630E-08	-0.28313017143611E-07	0.16881963498765E-08	-0.25784672761512E-07
8	8	0.11158900910586E-08	-0.46193345511620E-08	0.41222683531745E-09	-0.38552091795683E-08

Table S13. | mJ > percentage composition of the 8 Kramers' doublets belonging to the fundamental $^6H_{15/2}$ multiplet for the endo-endo **Dy(TTA)** derivative. The quantization axis is the easy-axis of the computed fundamental Kramers' doublet.

| m_J >	E_0	E_1	E_2	E_3	E_4	E_5	E_6	E_7
| ±15/2 >	89.6	2.0	0.3	0.2	0.1	0.1	6.3	1.3

| ±13/2 >	0.1	0.2	1.7	49.7	28.2	2.2	2.7	15.2
| ±11/2 >	6.9	0.1	1.1	0.8	1.3	15.8	62.5	11.5
| ±9/2 >	0.5	0.1	0.4	8.4	11.5	2.5	13.3	63.2
| ±7/2 >	0.8	1.1	1	7.5	17	50.8	14	7.8
| ±5/2 >	0.1	7.0	17.3	20.6	36.3	16.7	1.1	0.7
| ±3/2 >	0.7	27.4	53.7	6.5	2.1	9.5	0.1	0.1
| ±1/2 >	1.2	62.1	24.4	6.3	3.5	2.3	0.1	0.1

Table S14. Energy levels' splitting of the 8 Kramers' doublets belonging to the fundamental $^6H_{15/2}$ multiplet for **Dy$_{(BTA)}$**. All energies are reported in cm^{-1}.

Kramers' Doublet	*Dy$_{(TTA)}$*
E$_0$	0.0000000
E$_1$	17.58351708
E$_2$	95.28412091
E$_3$	117.08012464
E$_4$	166.43292395
E$_5$	205.29775535
E$_6$	220.82426855
E$_7$	297.17977799

Table S15. Computed g-tensors and directions of the main magnetic axes for the ground and first excited Kramers' doublets of **Dy$_{(BTA)}$**.

Dy$_{(BTA)}$					
**Kramers' doublet**	**Component**	**Value**	**a**	**b'**	**c***
E$_0$	***g_x***	0.21040900	0.249294	-0.791184	-0.558463
	g_y	0.39183557	0.968351	0.210902	0.133476
	g_z	19.25447351	0.012177	-0.574063	0.818721
E$_1$	***g_x***	0.02244395	0.993256	-0.029183	0.112208
	g_y	0.06183427	0.107474	0.594809	-0.796650
	g_z	19.42444005	-0.043493	0.803337	0.593934

Table S16. Extended Stevens' Operators (ESOs) B_k^q coefficients mapped on the fundamental $^6H_{15/2}$ multiplet for the **Dy$_{(BTA)}$** derivative. The quantization axis is the easy axis of the computed fundamental Kramers' doublet.

k	q	Dy$_{(BTA)}$
2	-2	0.17273050169945E+00
2	-1	-0.47354997295215E+00
2	0	-0.28338269484018E+00
2	1	0.12310653515493E+01
2	2	-0.20558175340778E+00
4	-4	0.90079807783096E-02
4	-3	-0.10658921707157E-01
4	-2	-0.95094727752062E-02
4	-1	0.11938316784843E-02
4	0	-0.50370701061945E-02
4	1	-0.35389794912909E-04

4	2	0.25455422593452E-01
4	3	0.92689328524669E-02
4	4	-0.12030197059803E-01
6	-6	-0.22090370263333E-03
6	-5	-0.14247811316506E-03
6	-4	-0.72516690049842E-04
6	-3	-0.54709473159876E-04
6	-2	-0.12015066474466E-05
6	-1	0.25257720027703E-04
6	0	-0.78751775123236E-05
6	1	-0.71127924934543E-04
6	2	0.10966113214941E-04
6	3	0.49008588395337E-04
6	4	0.75007239933601E-04
6	5	0.10721137978288E-04
6	6	0.19004211487009E-03
8	-8	0.28524304356657E-07
8	-7	-0.51776975361302E-07
8	-6	-0.77788799858345E-07
8	-5	-0.14595583369104E-06
8	-4	0.12001134359961E-08
8	-3	0.35880397676319E-07
8	-2	0.48223608360948E-07
8	-1	-0.43512245238868E-09
8	0	0.33609755467720E-08
8	1	-0.15517160391971E-08
8	2	-0.16982914422923E-06
8	3	0.24867155462832E-08
8	4	-0.18716862694787E-08
8	5	0.37201542078206E-08
8	6	0.80761801954226E-07
8	7	-0.34089429278845E-08
8	8	-0.12303591237338E-07

Table S17. $|m_J\rangle$ percentage composition of the 8 Kramers' doublets belonging to the fundamental $^6H_{15/2}$ multiplet for the **Dy(BTA)** derivative. The quantization axis is the easy-axis of the computed fundamental Kramers' doublet.

$\| m_J >$	E_0	E_1	E_2	E_3	E_4	E_5	E_6	E_7
| ±15/2 >	89.3	0.2	1	1.1	2.7	2	0.6	3
| ±13/2 >	0	0.6	38.9	8.7	22.7	8.2	4.6	16.3
| ±11/2 >	9.4	0.7	1.5	5.2	14.3	24.5	10.9	33.5
| ±9/2 >	0	0.3	32.8	3.8	3.3	8.4	20.1	31.3
| ±7/2 >	0.2	3.8	4.7	44.8	1	11.4	21.9	12.3
| ±5/2 >	0.1	16.3	5.9	33.5	7.1	18.6	17.1	1.5
| ±3/2 >	0.6	33.3	8.9	1.8	35.8	4.9	13.4	1.5
| ±1/2 >	0.2	44.7	6.3	1.3	13.3	22	11.6	0.6

10. References

(1) Teotonio, E. E. S.; Silva, F. A.; Pereira, D. K. S.; Santo, L. M.; Brito, H. F.; Faustino, W. M.; Felinto, M. C. F. C.; Santos, R. H.; Moreno-Fuquen, R.; Kennedy, A. R.; Gilmore, D. Luminescence Enhancement of the Tb(III) Ion with the Thenoyltrifluoroacetonate Ligand Acting as an Efficient Sensitizer. *Inorg. Chem. Commun.* **2010**, *13* (12), 1391–1395. https://doi.org/10.1016/j.inoche.2010.07.043.

(2) Teotonio, E. E. S.; Fett, G. M.; Brito, H. F.; Faustino, W. M.; De Sá, G. F.; Felinto, M. C. F. C.; Santos, R. H. A. Evaluation of Intramolecular Energy Transfer Process in the Lanthanide(III) Bis- and Tris-(TTA) Complexes: Photoluminescent and Triboluminescent Behavior. *J. Lumin.* **2008**, *128* (2), 190–198. https://doi.org/10.1016/j.jlumin.2007.07.005.

(3) Bukvetskii, B. V.; Mirochnik, A. G.; Zhikhareva, P. A.; Karasev, V. E. Crystal Structure and Triboluminescence of the [Eu(TTA)2(NO3)(TPPO)2] Complex. *J. Struct. Chem.* **2006**, *47* (3), 575–580. https://doi.org/10.1007/s10947-006-0340-6.

(4) Mirochnik, A. G.; Bukvetskii, B. V.; Zhikhareva, P. A.; Polyakova, N. V.; Karasev, V. E. Crystal Structure and Triboluminescence of the [Tb(BTFA)2(NO3)(TPPO)2] Complex. *Russ. J. Inorg. Chem.* **2006**, *51* (5), 737–742. https://doi.org/10.1134/S003602360605010X.

(5) Sheldrick, G. M. SADABS-Program for Empirical Absorption Correction of Area Detector Data, 1996.

(6) Sheldrick, G. M. *SHELXT* – Integrated Space-Group and Crystal-Structure Determination. *Acta Crystallogr. Sect. Found. Adv.* **2015**, *71* (1), 3–8. https://doi.org/10.1107/S2053273314026370.

(7) Sheldrick, G. M. Crystal Structure Refinement with *SHELXL*. *Acta Crystallogr. Sect. C Struct. Chem.* **2015**, *71* (1), 3–8. https://doi.org/10.1107/S2053229614024218.

(8) Llnunell, M.; Casanova, D.; Cirera, J.; Alemany, P.; Alvarez, S. SHAPE Program for the Stereochemical Analysis of Molecular Fragments by Means of Continuous Shape Measures and Associated Tools, 2013.

(9) Spek, A. L. PLATON, An Integrated Tool for the Analysis of the Results of a Single Crystal Structure Determination. *Acta Crystallogr. A* **1990**, *46* (s1), 34. https://doi.org/10.1107/S0108767390099780.

(10) Farrugia, L. J. WinGX Suite for Small-Molecule Single-Crystal Crystallography. *J. Appl. Crystallogr.* **1999**, *32* (4), 837–838. https://doi.org/10.1107/S0021889899006020.

(11) Brandenburg, K. Diamond - Crystal and Molecular Structure Visualization, Crystal Impact GbR, 2014. https://www.crystalimpact.com/diamond/ (accessed 2024-09-27).

(12) Macrae, C. F.; Edgington, P. R.; McCabe, P.; Pidcock, E.; Shields, G. P.; Taylor, R.; Towler, M.; Streek, J. van de. Mercury: Visualization and Analysis of Crystal Structures. *J. Appl. Crystallogr.* **2006**, *39* (3), 453–457. https://doi.org/10.1107/S002188980600731X.

(13) Kitos, A. A.; Gálico, D. A.; Mavragani, N.; Castañeda, R.; Moilanen, J. O.; Brusso, J. L.; Murugesu, M. Probing Optical and Magnetic Properties via Subtle Stereoelectronic Effects in Mononuclear DyIII-Complexes. *Chem. Commun.* **2021**, *57* (63), 7818–7821. https://doi.org/10.1039/D1CC02407H.

(14) Mattei, C. A.; Delouche, T.; Lefeuvre, B.; Dallon-Cordier, M.; Lalli, C.; Cador, O.; Abad Galán, L.; Riobé, F.; Maury, O.; Bouit, P.-A.; Pointillart, F. Luminescent and Sublimable Binaphthyl-Based Field-Induced Lanthanide Single-Molecule Magnets. *Chem. Squared* **2022**, *6*, 1. https://doi.org/10.28954/2022.csq.06.001.

(15) Neese, F.; Wennmohs, F.; Becker, U.; Riplinger, C. The ORCA Quantum Chemistry Program Package. *J. Chem. Phys.* **2020**, *152* (22), 224108. https://doi.org/10.1063/5.0004608.

(16) Aravena, D.; Neese, F.; Pantazis, D. A. Improved Segmented All-Electron Relativistically Contracted Basis Sets for the Lanthanides. *J. Chem. Theory Comput.* **2016**, *12* (3), 1148–1156. https://doi.org/10.1021/acs.jctc.5b01048.

(17) Weigend, F.; Ahlrichs, R. Balanced Basis Sets of Split Valence, Triple Zeta Valence and Quadruple Zeta Valence Quality for H to Rn: Design and Assessment of Accuracy. *Phys. Chem. Chem. Phys.* **2005**, *7* (18), 3297–3305. https://doi.org/10.1039/B508541A.

(18) Neese, F.; Wennmohs, F.; Hansen, A.; Becker, U. Efficient, Approximate and Parallel Hartree–Fock and Hybrid DFT Calculations. A 'Chain-of-Spheres' Algorithm for the Hartree–Fock Exchange. *Chem. Phys.* **2009**, *356* (1), 98–109. https://doi.org/10.1016/j.chemphys.2008.10.036.

(19) Chibotaru, L. F.; Ungur, L. Ab Initio Calculation of Anisotropic Magnetic Properties of Complexes. I. Unique Definition of Pseudospin Hamiltonians and Their Derivation. *J. Chem. Phys.* **2012**, *137* (6), 064112. https://doi.org/10.1063/1.4739763.

(20) Ungur, L.; Chibotaru, L. F. Ab Initio Crystal Field for Lanthanides. *Chem. – Eur. J.* **2017**, *23* (15), 3708–3718. https://doi.org/10.1002/chem.201605102.
